# An alternative splicing caused by a natural variation in *BnaC02.VTE4* gene affects vitamin E and glucosinolate content in rapeseed (*Brassica napus* L.)

**DOI:** 10.1111/pbi.14603

**Published:** 2025-02-04

**Authors:** Furong Wang, Lieqiong Kuang, Zelin Xiao, Ze Tian, Xinfa Wang, Hanzhong Wang, Xiaoling Dun

**Affiliations:** ^1^ Key Laboratory of Biology and Genetic Improvement of Oil Crops, Oil Crops Research Institute of the Chinese Academy of Agricultural Sciences Ministry of Agriculture and Rural Affairs Wuhan China; ^2^ Tianshui Institute of Agricultural Sciences Tianshui China; ^3^ Hubei Hongshan Laboratory Wuhan China

**Keywords:** rapeseed, vitamin E, alpha‐tocopherol, glucosinolate, *VTE4*

## Abstract

Vitamin E (VE) is essential for plants and animals. Rapeseed oil is rich in α‐tocopherol (α‐T), which is the most bioactive form of VE in human body. This study demonstrated that VE in rapeseed seeds was mainly controlled by embryo genotype through incomplete diallel hybridization. By genome‐wide association study, the QTL‐qVE.C02 associated with VE and α‐T contents was detected in a *Brassica napus* association population, and the phenotypic contribution rate was up to 18.71%. *BnaC02.VTE4*, encoding gama‐tocopherol methyltransferase, was proved as the target gene of qVE.C02 by genetic complementation. Two *BnaC02.VTE4* haplotypes were identified in the population. Compared with *BnaC02.VTE4* ^HapH^, a point mutation from A to G at the 3′ splicing site of the second intron were found in *BnaC02.VTE4* ^HapL^, resulting in alternative splicing and early termination of translation. HapL^1052(G‐A)^, the site‐directed mutagenesis fragment of *BnaC02.VTE4* ^HapL^, was introduced into *Arabidopsis vte4* mutant and 8S088 (a *BnaC02.VTE4* ^HapL^ accession), and the contents of VE and α‐T in *atvte4‐4* and 8S088 seeds were increased by 90.10% to 307.29%. These demonstrated the point mutation as the causal for the difference in VE biosynthesis in rapeseed. Further, this variation also led to the significant difference in glucosinolate content between *BnaC02.VTE4* ^HapH^ and *BnaC02.VTE4* ^HapL^ accessions. Multi‐omics analysis suggested that the expression of some genes and the accumulation of several metabolites related to the glucosinolate biosynthesis pathway were significantly increased in *BnaC02.VTE4* ^HapL^ group. Moreover, by functional marker identification, the *BnaC02.VTE4* ^HapH^ was found to be selected during domestication. Our findings offered promising opportunities for enhancing rapeseed quality traits.

## Introduction

Vitamin E (VE) is a generic term for tocopherols and tocotrienols and their derivatives. There are eight types of VE known in nature, including α, β, γ, δ‐tocopherol and α, β, γ, δ‐tocotrienol (Esteban *et al*., 2009; Kiyose, 2021; Muñoz and Munné‐Bosch, 2019). Among them, α‐tocopherol (α‐T) has the highest bioactivity in human body (Fanali *et al*., 2013; Kono *et al*., 2013). As a fat‐soluble nutrient indispensable for the survival of humans and animals, VE cannot be synthesized autonomously in non‐photosynthetic organisms. It contributes to the neutralization of free radicals, offering protection against oxidative stress, thus playing a crucial part in the prevention of numerous diseases as well as the preservation of overall health, such as immunomodulatory (Lee and Han, 2018), improving male fertility (Zhou *et al*., 2022), anti‐inflammation (Meydani *et al*., 2018), preventing cancer (de Sousa Coelho *et al*., 2023; Jiang, 2022) and preventing or delay Chronic diseases (Cerqua *et al*., 2022).

In plants, VE also has a variety of functions, such as delaying oxidative deterioration of vegetable oils (Alsamadany and Ahmed, 2022; Munné‐Bosch and Alegre, 2002), protecting plants from photoinhibition and photooxidation stress (Muñoz *et al*., 2018), improving seed storage tolerance and germination rate (Lee *et al*., 2020), participating in the regulation of plant senescence and signal transduction processes (Fang *et al*., 2019; Hofius and Sonnewald, 2003; Munné‐Bosch, 2019). In addition, VE can also improve crop resistance to a variety of abiotic stresses (Hasanuzzaman *et al*., 2014) such as salinity, heavy metals (Upadhyaya *et al*., 2021), drought (Ma *et al*., 2020) and cold (Zhang *et al*., 2020a). For example, increased VE content enhanced the tolerance of tobacco to salt and heavy metal stress by reducing ROS, lipid peroxidation and ion leakage (Jin and Daniell, 2014). The distribution of VE exists in different tissues of higher plants such as roots, stems (Crowell *et al*., 2008; Ji *et al*., 2016), leaves (Maeda *et al*., 2006), flowers (Muñoz *et al*., 2018), fruits (Gramegna *et al*., 2019) and seeds (Schuy *et al*., 2019). Among them, the content of VE in oilseed is the most abundant, and vegetable oil is the main source of VE for human (Gilliland *et al*., 2006; Siles et al., 2018).

Finding out the pathway of VE biosynthesis is the theoretical basis for breeding oil varieties with high VE content. Currently, shikimate (SK) pathway, methylerythritol phosphate (MEP) pathway and an additional chlorophyll degradation pathway are the major VE biosynthesis pathways that have been discovered (Muñoz and Munné‐Bosch, 2019; Zhang *et al*., 2015). SK pathway provides tyrosine as the precursor, and tyrosine aminotransferase (*TAT*) use tyrosine to generate 4‐Hydroxyphenyl‐pyruvate, further utilized by Hydroxyphenylpyruvate dioxygenase (*HPPD*) synthesis homogentisate (HGA). MEP pathway and chlorophyll degradation pathway generate phytyl diphosphate (PDP) under the action of geranylgeranyl diphosphate reductase (*GGDR*), phytyl kinase (*VTE5*), phytyl phosphate kinase (*VTE6*) and alpha/beta hydrolase (*VTE7*) (Aksoz *et al*., 2020; Albert et al., 2022; Kimura *et al*., 2018; Matthäus *et al*., 2021; Valentin *et al*., 2006). The hydrophilic head of VE is mainly synthesized by SK pathway using HGA as a precursor (Yang *et al*., 2011), and the hydrophobic tail is synthesized by MEP pathway using PDP and geranylgeranyl diphosphate (GGDP) as the precursors. *HPPD* and *GGDR* are the key rate‐limiting enzymes in the SK pathway and MEP pathway, respectively. While tocopherol cyclase (*VTE1*), homogentisate phytyl transferase (*VTE2*), methylphytylbenzoquinol methyltransferase (*VTE3*) and gamma‐tocopherol methyltransferase (*VTE4*) act together in the two pathways (Zhang *et al*., 2013). *VTE2* converts HGA and PDP into methylphytylbenzoquinol (MPBQ). Subsequently, MPBQ is converted into dimethylphytylbenzoquinol (DMPBQ) by *VTE3*, MPBQ and DMPBQ further utilized by *VTE1* generate δ‐T and γ‐T. Ultimately, *VTE4* converts γ‐T and δ‐T into α‐T and β‐T. In the process, SAM supplies methyl groups to *VTE3* and *VTE4* (Fritsche *et al*., 2017; Guo *et al*., 2022; Mène‐Saffrané and DellaPenna, 2010). According to the gene expression and function differences in these VE‐related pathways, the composition and content of VE are different in various oil crops (Aksoz *et al*., 2020; Matthäus *et al*., 2021).

Some fascinating interconnections in plant metabolic pathways are uncovered. For instance, tyrosine and methionine, which crucial in the biosynthesis of VE, are also essential amino acid precursors in the glucosinolate biosynthesis pathway. Under the catalysis of UGT74B1 (UDP‐Glucosyl Transferase 74B1) and other enzymes (Marroun *et al*., 2016), tyrosine is converted into 2‐Phenylethyl glucosinolate. Through a series of actions by genes such as *CYP83A1* (cytochrome P450 A1) (Bak and Feyereisen, 2001), *MAM1* (methylthioalkylmalate synthase 1) (Textor *et al*., 2004), *leuC* (3‐isopropylmalate dehydratase subunit) (Kirino *et al*., 1994), *P45083B1* (cytochrome P450 83B1) (Kim *et al*., 2015), *CYP79F1* (cytochrome p450 79F1) (Sharma *et al*., 2016) and *UGT74B1*, etc., methionine ultimately synthesizes various glucosinolate components, including 3‐Methylthiopropyl glucosinolate, 4‐Methylthiobutyl glucosinolate and so on. This interdependence among plant metabolic pathways indicates a potential link between the VE biosynthesis pathway and other metabolites biosynthesis pathways, such as the glucosinolate biosynthesis pathway.

Bioengineering is an important means to improve the total amount or quantity of VE in crops. For instance, overexpressing of *HPPD* in cyanobacteria increased the level of tocopherol by seven times. Overexpression of *VTE2* in *Arabidopsis* enhanced the tocopherol content in seeds by 60%. Similarly, overexpression of *VTE4* in maize boosted the α‐T content in seeds by 6.5 times and has resulted in a remarkable increase in the α‐/γ‐tocopherol ratio (Zhang *et al*., 2020b). Overexpressed *VTE1*, *VTE2* and *VTE1* + *VTE2* in Indica rice increased α‐T content in seeds by 2.7 times, 3.3 times and 4.1 times, respectively (Sundararajan *et al*., 2021). Furthermore, overexpression of *VTE4* in rapeseed not only increased seed tocopherol and fatty acid contents, but also enhanced the salt tolerance during germination (Guo *et al*., 2022).

Rapeseed is the third oil crop in the world and the largest oil crop in China. Unlike peanut oil and soybean oil, rapeseed oil is highly enriched in α‐T (Matthaus *et al*., 2016). It is of great significance to breed rapeseed varieties with high VE content, and thus to improve their stress resistance and edible quality by revealing the mechanism of VE biosynthesis in rapeseed and excavating excellent alleles. In this study, we demonstrated that *BnaC02.VTE4* was the target gene of qVE.C02 by genome‐wide association study (GWAS) and genetic complementation test in *Brassica napus*. The single base mutation of *BnaC02.VTE4* in the natural population produced two alternative splicing transcripts with functional domain deletion, which hindered the conversion of γ‐tocopherol (γ‐T) to α‐T, thus impaired the accumulation of tocopherol and significantly affected the metabolites related to the glucosinolate biosynthesis pathway.

## Results

### VE content in rapeseed seeds mainly controlled by embryo genotype

**Figure 1:**
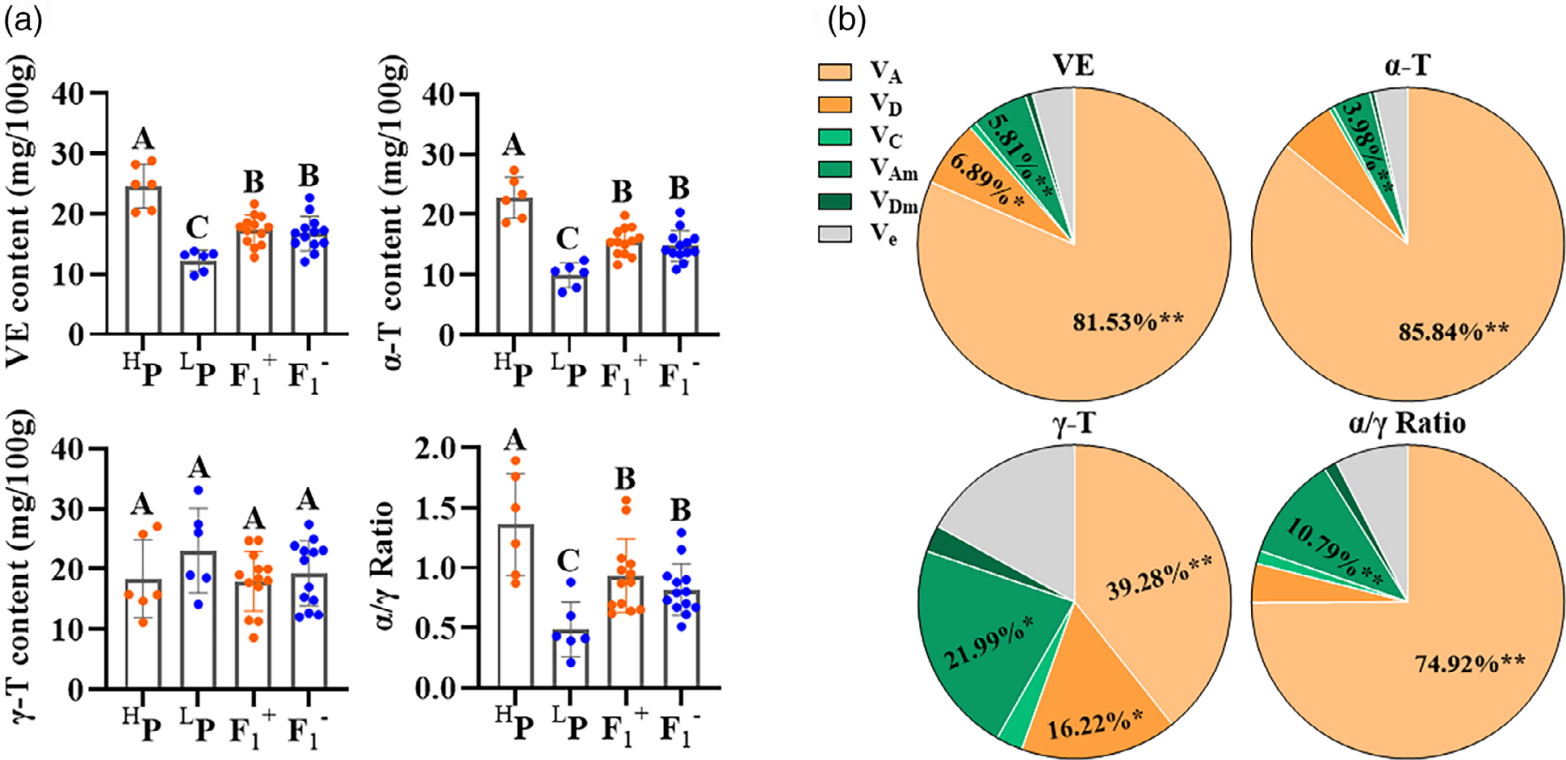
Genetic Analysis of VE‐related traits in rapeseed seeds. (a) The comparison of VE content and its components in seeds of ^H^P and ^L^P rapeseed inbred lines and their F_1_ reciprocal hybrids. ^H^P and ^L^P included six parents with high and low VE contents, respectively. F_1_^+^ and F_1_^−^ included 13 F_1_ generation with ^H^P and ^L^P as the female parent, respectively. A, B and C represented the significant differences (*P* < 0.01). (b) Estimation of genetic variance components for VE content in rapeseed seeds. V_A_ and V_D_, embryo additive and dominance variances. V_C_, cytoplasmic variance. V_Am_ and V_Dm_, maternal additive and dominance variances. V_e_, residual variance. *, significant differences with *P* < 0.05. **, *P* < 0.01.

In rapeseed, VE is mainly composed of α‐T and γ‐T (Wang *et al*., 2012; Xu *et al*., 2019). In order to reveal the genetic preference of the contents of VE and its components in *B. napus*, an incomplete diallel hybridization (NCII) was performed on six germplasms with high VE content (H01‐H06) and six germplasms with low VE content (L01‐L06), and a total of 13 hybrid combinations were generated (Table S1). Detection of the contents of VE and its components in seeds of high VE parents (^H^P), low VE parents (^L^P) and F_1_ generation showed that VE traits did not differ significantly between reciprocal crosses F_1_^+^ (^H^P × ^L^P) and F_1_^−^ (^L^P × ^H^P) (Table S1 and Figure 1a). The VE and α‐T contents, and the α/γ ratio of hybrids were significantly different from those of their parents (*P* < 0.01), while there was no significant difference in γ‐T among the parents and hybrids (Figure 1a). The mid‐parent heterosis distribution of VE content was ranged from −36.62% to 19.34%, with the mean mid‐parent heterosis for orthogonal and inverse crosses being −4.62% and −7.19% (Table S2), indicating that VE content in seeds of rapeseed generally did not possess hybrid vigour.

QGA2.0 software (http://ibi.zju.edu.cn/software/qga/) was used to calculate the diploid seed embryo‐cytoplasm‐maternal effect (2nGoCGm model). It was found that the embryo additive effect (V_A_) of VE, α‐T and γ‐T contents, and α/γ ratio of rapeseed seeds were 81.5%, 85.84%, 39.28% and 74.92%, respectively. The maternal genotype effects (additive + dominant) were 6.53%, 4.47%, 24.74% and 12.13%, respectively (Figure 1b). These results indicated that the VE content in rapeseed seeds was mainly controlled by the embryo genotype, with additive effects being predominant and there was a weak maternal additive effect. Therefore, in the breeding of hybrid varieties of rapeseed with high VE content, it is necessary to improve the VE content of both parents simultaneously.

### Identification of VE‐associated QTLs using GWAS

**Figure 2:**
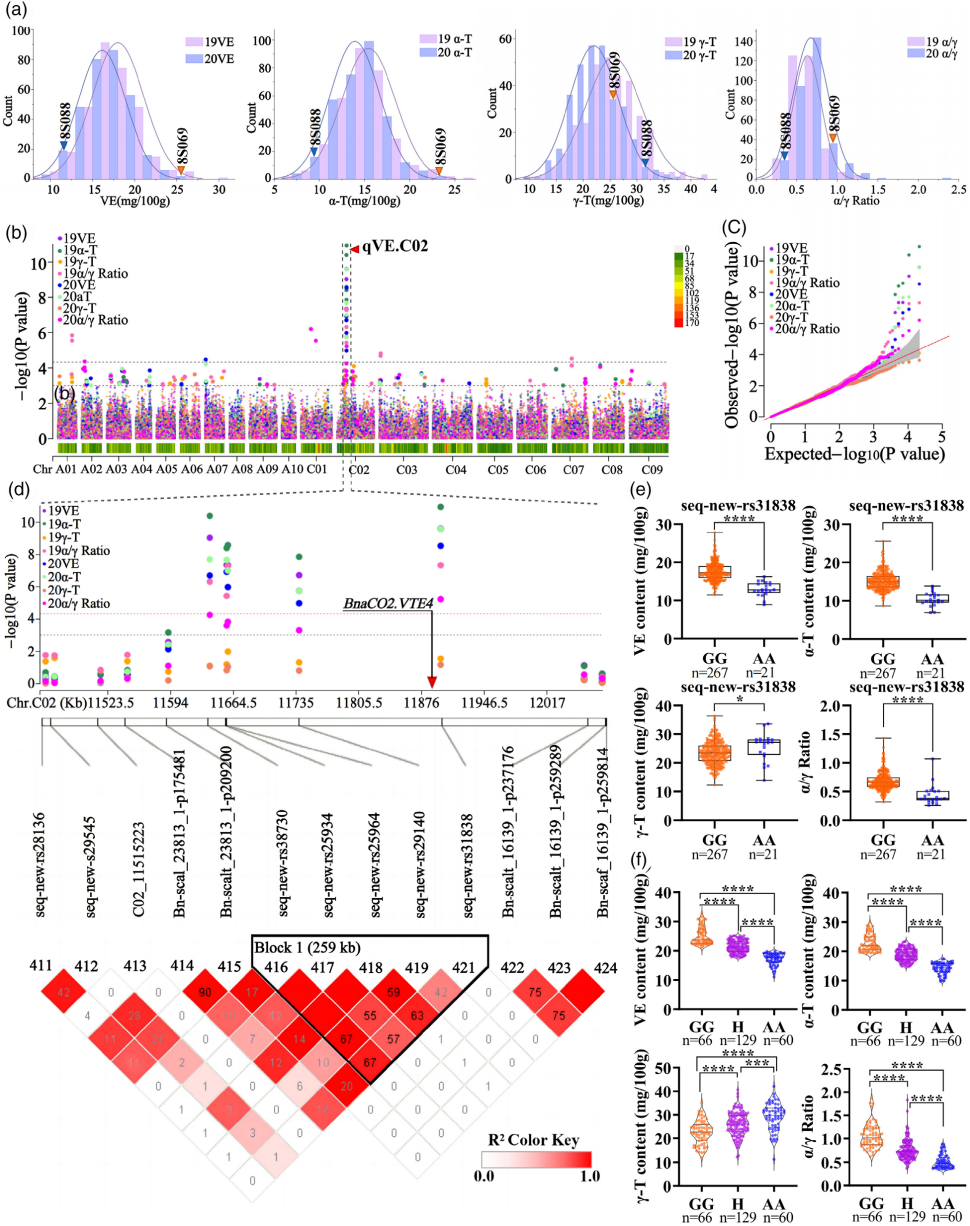
GWAS for the VE‐related traits in *B. napus*. (a) Frequency distribution of the VE, α‐T and γ‐T contents, and α/γ ratio under two consecutive years. Arrows represented the location of 8S088 and 8S069 in the population based on their phenotypes. (b) Manhattan plots for VE‐related traits using MLM. (c) Quantile‐quantile plots for VE‐related traits. (d) Local manhattan plot and LD heatmap surrounding qVE.C02. The part enclosed in black box represented the candidate region. (e) Comparative analyses of VE‐related traits between the two haplotypes of SNP seq‐new‐rs31838 at qVE.C02. (f) Genotyping and phenotyping of seq‐new‐rs31838 in the F_2_ segregating population from the cross between 8S069 and 8S088. *, *** and **** indicated significant differences at the level of *P* < 0.05, *P* < 0.001 and *P* < 0.0001, respectively.

To further reveal the genetic mechanism of VE‐related traits in rapeseed and to identify regulatory loci, the contents of VE and its components in seeds were determined in a *B. napus* association population consisting of 327 accessions under field conditions for two consecutive years (2019 and 2020). Statistical analysis indicated that the seed VE, α‐T and γ‐T contents, and α/γ ratio in the population were approximately normal distributions (Figure 2a). The coefficients of variation (CV) were 4.76%–20.53%, and the heritabilities (*h* ^2^) were 0.69–0.83 (Table S3), indicating that genetic variation was the main caucal of phenotypic variation.

The 50K Illumina Infinium SNP chip, including 45 708 SNP markers, was used for genotyping the population. After screening, a total of 20 131 SNP markers were obtained for trait‐SNP association analysis (Ibrahim *et al*., 2021). Using a mixed linear model (MLM), GWAS analysis identified a total of 24 marker‐trait associations SNPs with a significant threshold of −log_10_^(*P*)^ > 4.30 (Table S4). Important SNPs associated with studied traits were shown on both Manhattan and QQ plots (Figure 2b,c). SNPs that were genetically close (within 1 MB) or had a high linkage disequilibrium (LD) with *R* ^2^ > 0.2 were considered to be a QTL cluster (Liu *et al*., 2016). Ultimately, these SNPs were consolidated into four QTL clusters (Table S4), Among them, qVE.C02 including five SNPs associated with VE and α‐T contents, were repeatedly detected within 2 years and the phenotypic variation explained rate (PVE) was 8.74%–18.71% (Table S4 and Figure 2d).

For qVE.C02, the GG haplotype of the SNP seq‐new‐rs31838 with the highest PVE showed significantly higher VE and α‐T contents, and α/γ ratio compared to the AA haplotype in the natural population, while the γ‐T content was opposite (Figure 2e). Further, an F_2_ segregating population including 255 individual plants through the hybridization of 8S069 (H06) and 8S088 (L03) were genotyped by seq‐new‐rs31838 (Figure 2f). In the F_2_ population, VE and α‐T contents, and α/γ ratio showed a pattern of GG haplotype > heterozygotes > AA haplotype, while the γ‐T content was opposite (*P* < 0.01). This further verified the additive effect of qVE.C02 on the regulation of VE biosynthesis, especially the biosynthesis of α‐T in rapeseed seeds.

### Cloning and validation of the target gene of qVE.C02

Haplotype analysis suggested that the LD Block interval of qVE.C02 ranged from 11 638 002 to 11 897 577, with a total of 259.6 kb according to the Darmor‐Bzh genome (Chalhoub *et al*., 2014). There were 29 annotated genes in this interval (Table S5), among which *BnaC02g16270D* encodes gamma‐tocopherol methyltransferase (γ‐TMT) and is 1530 bp away from seq‐new‐rs31838. Based on its functional annotation, it was renamed *BnaC02.VTE4*.

**Figure 3:**
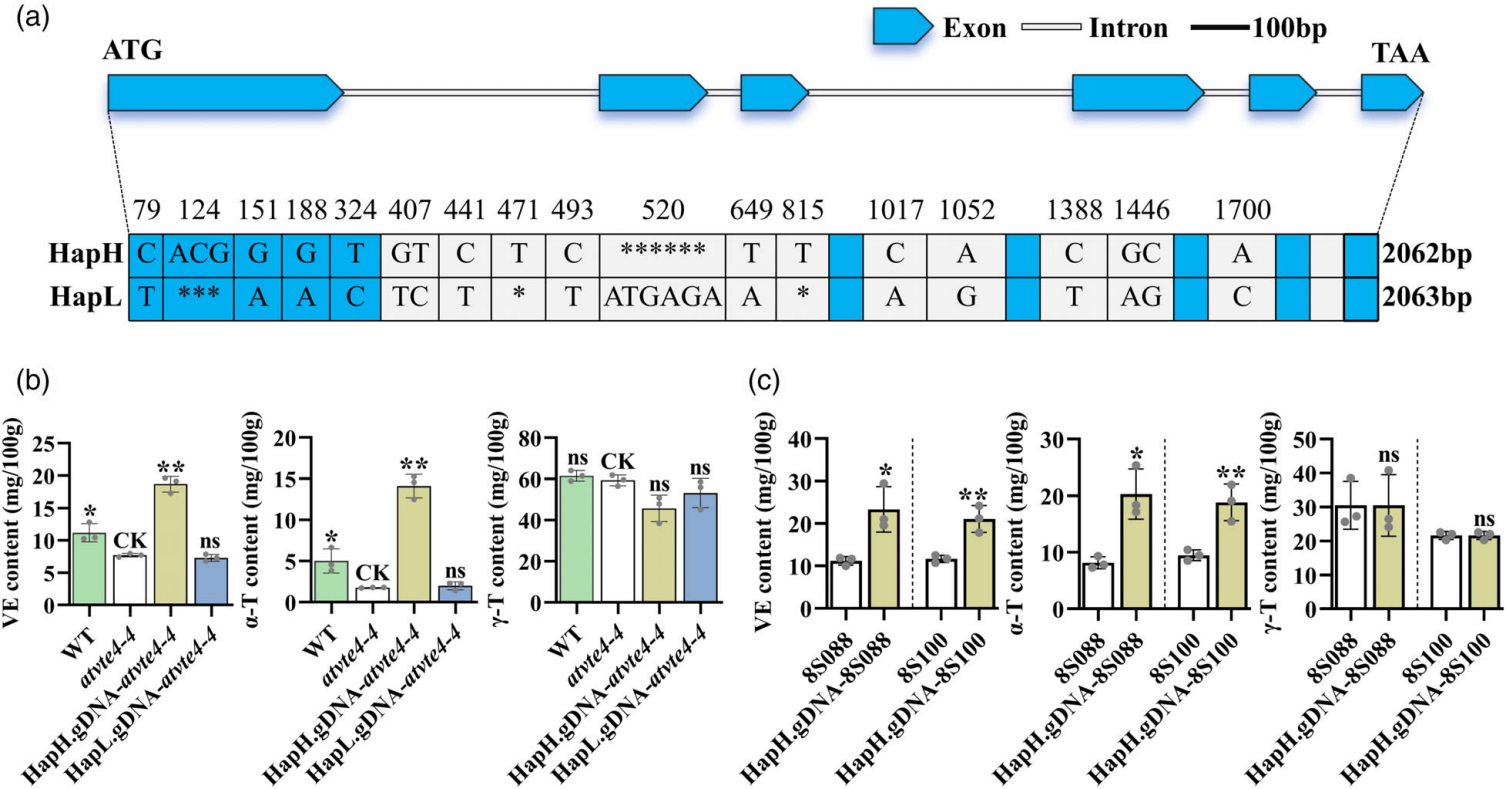
Identification and characterization of *BnaC02.VTE4*. (a) Sequence variations between *BnaC02.VTE4* ^*HapH*^ and *BnaC02.VTE4* ^*HapL*^. Variations were displayed below with their location away from the start codon. *, ***, ****** indicated that the corresponding base was missing. (b) Comparison of VE content in seeds of *Arabidopsis thaliana* Col‐0 (WT), *atvte4‐4*, *atvte4‐4* heterologously introduced with the full‐length gDNA of *BnaC02.VTE4* ^*HapH*^ and *BnaC02.VTE4* ^*HapL*^. (c) Phenotype of *BnaC02.VTE4* ^*HapH*^ complementary lines of the *BnaC02.VTE4* ^*HapL*^ genotypes, 8S088 and 8S100. All values were presented as mean ± SD with significant difference displayed by *P < 0.05 and **P < 0.01.

Two haplotypes, *BnaC02.VTE4* ^*HapH*^ and *BnaC02.VTE4* ^*HapL*^, were identified by the genomic sequence analysis of *BnaC02.VTE4* among 20 accessions, respectively. Compared with *BnaC02.VTE4* ^*HapH*^, *BnaC02.VTE4* ^*HapL*^ displayed 31 variations in 2200 bp upstream of the start codon, 4 SNPs and 3 bp deletion in the first exon, 13 SNPs and 6 bp insertion in the introns, and 3 variations in the 600 bp downstream of the TAA stop codon (Figure 3a and Figure S1). No significant changes in the predicted cis‐acting elements within 2200 bp upstream of the start codon (Figure S2).

The full‐length gDNA constructs of *BnaC02.VTE4* ^*HapH*^ and *BnaC02.VTE4* ^*HapL*^ were introduced into the *atvte4‐4* (SALK_036736C), a mutant of the *Arabidopsis* homologous gene that is typical defective in α‐T biosynthesis. Compared with *atvte4‐4*, the contents of VE and α‐T in seeds of the *BnaC02.VTE4* ^*HapH*^ transgenic *atvte4‐4* lines were significantly increased, while no difference in the *BnaC02.VTE4* ^*HapL*^ transgenic lines (Figure 3b). Subsequently, the full‐length gDNA constructs of *BnaC02.VTE4* ^*HapH*^ was introduced into the low VE germplasms with the *BnaC02.VTE4* ^*HapL*^ genotype (8S088 and 8S100). Compared to the negative controls of 8S088 and 8S100, the VE content in the complementary lines significantly increased with 108.21% and 81.01%, respectively, mainly due to the significant increase in α‐T content (Figure 3c). Thus, the sequence variation of *BnaC02.VTE4* results in VE content difference in the natural population, which mainly affects the biosynthesis of α‐T.

### The alternative splicing of *BnaC02.VTE4* caused by a point mutation

**Figure 4:**
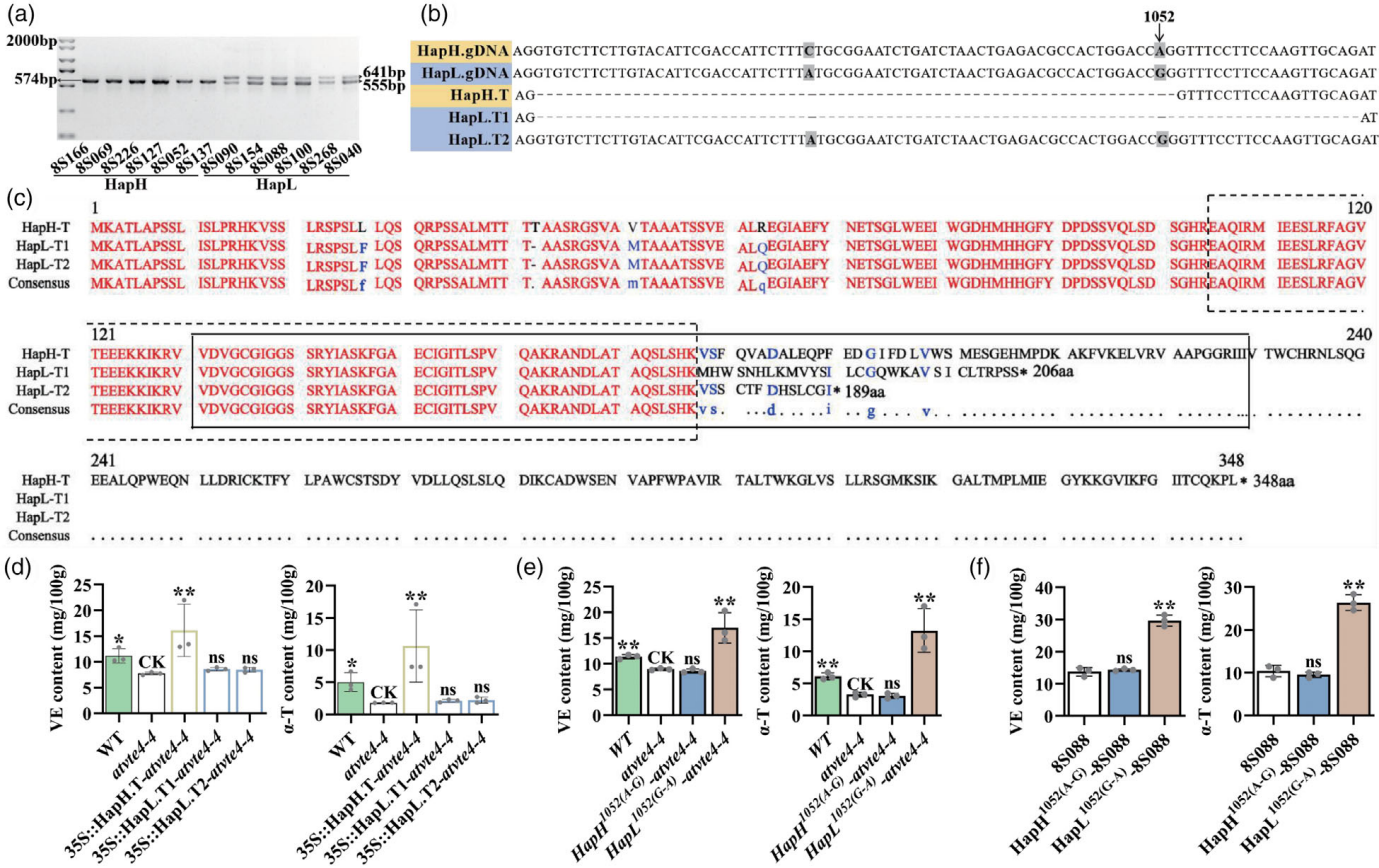
Identification of functional variants in *BnaC02.VTE4*. (a) The transcripts' fragment of *BnaC02.VTE4* ^*HapH*^ and *BnaC02.VTE4* ^*HapL*^ amplified from six high VE accessions (HapH) and six low VE accessions (HapL). (b) The comparison of gDNA and cDNA sequence of *BnaC02.VTE4* ^*HapH*^ and *BnaC02.VTE4* ^*HapL*^ from the second exon to the third exon. (c) The comparison of amino acid sequences of *BnaC02.VTE4* haplotypes. Conserved domain CMAS and Ubie_methyltran were enclosed by black dashed line and black solid line, respectively. (d) Comparison of the VE and α‐T contents in seeds of Col‐0, *atvte4‐4* and *atvte4‐4* heterologously introduced with *35Spro::HapH‐T*, *35Spro::HapL‐T1* and *35Spro::HapL‐T2*. (e) Comparison of the VE and α‐T content in seeds of Col‐0, *atvte4‐4* and *atvte4‐4* heterologously introduced with HapH^1052(A‐G)^ and HapL^1052(G‐A)^. (f) Comparison of the VE and α‐T contents in seeds of 8S088 and its transgenic lines introduced with HapH^1052(A‐G)^ and HapL^1052(G‐A)^. All data were presented as mean ± SD. CK, control; ns, no significant; *P < 0.05; **P < 0.01.

To further clarify the reason for the functional differences, the transcripts were cloned between the two haplotypes of *BnaC02.VTE4*. Interestingly, by PCR amplification, we found two sets of transcripts in the *BnaC02.VTE4* ^*HapL*^ germplasms, HapL‐T1 and HapL‐T2. Using the primer pEX14‐F/R (Table S6), the fragment of HapL‐T1 and HapL‐T2 were 641 bp and 555 bp, respectively. However, the fragment of *BnaC02.VTE4* ^*HapH*^ transcript (HapH‐T) was 574 bp (Figure 4a). Further analysis of the cause of alternative splicing in *BnaC02.VTE4* ^*HapL*^ revealed that the 3′ splicing site of the second intron at the 1052 bp after the start codon was mutated, changing from A to G (Figure 4b). This mutation resulted in two changes in the cleavage of the second intron (67 base pairs long) of *BnaC02.VTE4* ^*HapL*^: a sequence 19 bp longer than the original intron was cleaved (HapL‐T1) or the intron was retained (HapL‐T2) (Figure 4b), leading to early termination of the translation occurred in both transcripts of *BnaC02.VTE4* ^*HapL*^ (Figure 4c). Protein structure analysis of VTE4 homologues from different species suggested that VTE4 contains two conserved domains, CMAS (Cyclopropane‐fatty‐acyl‐phospholipid synthase) and Ubie_methyltran (Ma *et al*., 2019) (Figure S3). However, early termination in HapL‐T1 and HapL‐T2 resulted in the absence of 24 aa and 41 aa in their Ubie_methyltran domains, which could be the causal for the functional loss of *BnaC02.VTE4* ^*HapL*^ (Figure 4c).

Further, the expression vectors of *35Spro::HapH‐T*, *35Spro::HapL‐T1* and *35Spro::HapL‐T2*, were constructed and introduced into *atvte4‐4*. The results showed that only heterologous expression of *35Spro::HapH‐T* increased the VE and α‐T contents in *atvte4‐4* (Figure 4d). Subsequently, we constructed two site‐directed mutagenesis fragments of the *BnaC02.VTE4* genomic sequence, HapH^1052(A‐G)^ and HapL^1052(G‐A)^. HapH^1052(A‐G)^ was the mutation of *BnaC02.VTE4* ^*HapH*^ from A to G at 1052 bp, and HapL^1052(G‐A)^ caused *BnaC02.VTE4* ^*HapL*^ to mutate from G to A at 1052 bp. The fragments were both transformed into *atvte4‐4* and the *BnaC02.VTE4* ^HapL^ genotype 8S088. The results showed that the introduction of HapL^1052(G‐A)^ increased the contents of VE and α‐T in *atvte4‐4* seeds by 90.10% and 307.29%, meanwhile in 8S088 seeds by 116.17% and 153.93%, respectively (Figure 4e,f). However, the introduction of HapH^1052(A‐G)^ had no effect on these. The results proved that the mutation of A to G at the 3′ splicing site of the second intron in *BnaC02.VTE4* was the causal of the difference in VE and α‐T biosynthesis in rapeseed.

### Spatiotemporal expression patterns of *BnaC02.VTE4* and distribution of VE in rapeseed

**Figure 5:**
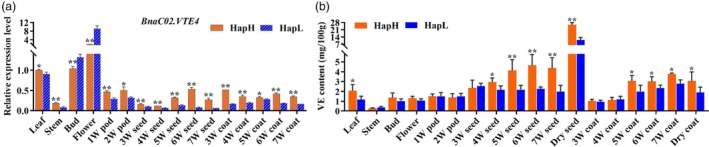
Differential analysis of expression of *BnaC02.VTE4* and VE accumulation in HapH and HapL genotypes. (a) Expression patterns of *BnaC02.VTE4* in different tissues of HapH and HapL. (b) VE accumulation in various tissues of HapH and HapL. HapH and HapL equally mixed the tissues of six *BnaC02.VTE4* ^*HapH*^ and *BnaC02.VTE4* ^*HapL*^ varieties, respectively. All data were presented as mean ± SD with 3 biological replicates. *P < 0.05; **P < 0.01.

Quantitative real time polymerase chain reaction (qRT‐PCR) showed that *BnaC02.VTE4* was expressed in multiple tissues of rapeseed, including leaf, stem, bud, flower, seed and pod coat, etc. It was highest expressed in flower, followed by flower bud (Figure 5a). The HapH (mix of six *BnaC02.VTE4* ^*HapH*^ materials) showed higher expression of *BnaC02.VTE4* throughout seed development than the HapL (mix of six *BnaC02.VTE4* ^*HapL*^ materials), and the difference reached largest at 6 weeks after pollination (6 W) with a ratio of 6.67 (Figure 5a). As shown in Figure 5b, VE exhibited differential distribution among various tissues and gradually increased during seed development until it reached the maximum accumulation in mature seeds. Moreover, VE content of *BnaC02.VTE4* ^*HapH*^ and *BnaC02.VTE4* ^*HapL*^ genotypes differed significantly only in leaves, seeds after 4 W and pod coats after 5 W, but not in stem, bud, flower. The difference of VE content between HapH and HapL was the largest in seed at 6 W with the ratio reached 2.04 times (Figure 5b). Based on the temporal and spatial expression patterns of *BnaC02.VTE4* and distribution of VE in rapeseed, it was concluded that 4 W to 6 W was the critical period for VE accumulation in seeds of rapeseed in this population.

### Multi‐omics analysis revealed the regulatory mechanism of *BnaC02.VTE4* in VE and glucosinolate biosynthesis

Transcriptome and metabolome analysis were performed on seeds at 4 W and 6 W of HapH (group‐H) and HapL (group‐L) for qRT‐PCR. For transcriptome data, principal component analysis (PCA) showed that L6WS (group‐L at 6 W) and H6WS (group‐H at 6 W) were significantly separated in PC1 and PC2 dimensions, accounting for 92.32% and 4.88% of the total variability, respectively, while there was no significant separation between L4WS (group‐L at 4 W) and H4WS (group‐H at 4 W) (Figure S4a). For metabolism data, L4WS and H4WS, L6WS and H6WS were significantly separated in PC1 and PC2, accounting for 64.16% and 8.57% of the total variability, respectively (Figure S4b). These results demonstrated the reliability of the omics data.

**Figure 6:**
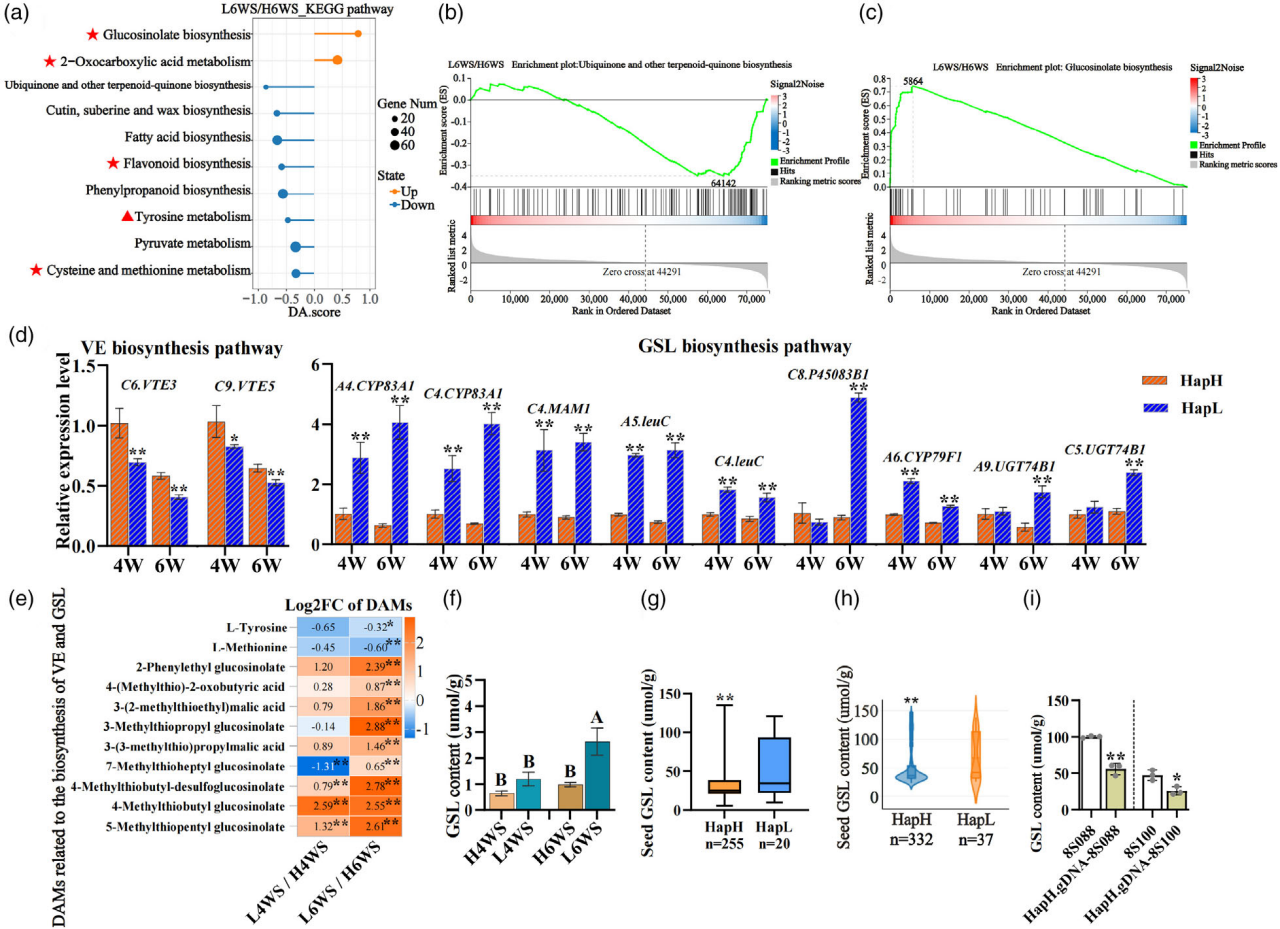
Transcriptome and metabolome analysis between *BnaC02.VTE4* ^*HapH*^ and *BnaC02.VTE4* ^*HapL*^ accessions. (a) KEGG pathway enrichment chart of L6WS/H6WS. DA.score calculation formula: (Up‐regulated DEGs – Down‐regulated DEGs)/Total DEGs. ★Both significantly enriched with DEGs and DAMs; ▲Only significantly enriched with DAMs. (b, c) Barcode enrichment silhouettes of ubiquinone and other terpenoid‐quinone biosynthesis pathway and glucosinolate biosynthesis pathway in L6WS/H6WS. (d) qRT‐PCR of several genes related to the biosynthesis of VE and glucosinolate. (e) Heatmap of the main DAMs related to the biosynthesis of VE and glucosinolate. (f) The comparsion of glucosinolate content in seeds between group‐H and group‐L at 4 W and 6 W. (g, h) The comparison of seed glucosinolate content in our association population and the germplasms from *Brassica napus* multi‐omics information resource (BnIR, https://yanglab.hzau.edu.cn/) carrying *BnaC02.VTE4* ^*HapH*^ and *BnaC02.VTE4* ^*HapL*^. (i) Detection of glucosinolate content in the *BnaC02.VTE4* ^*HapH*^ complementary lines of 8S088 and 8S100. *n* = 3, data were presented as mean ± SD. *P < 0.05; **P < 0.01.

Pairwise comparisons were conducted between group‐H and group‐L at each stage. A total of 5687 differentially expressed genes (DEGs) and 1655 differential metabolites (DAMs) were identified by the comparison of H4WS‐vs‐L4WS. Moreover, 6373 DEGs and 1756 DAMs were identified between H6WS‐vs‐L6WS (Figure S4c,d). Kyoto Encyclopedia of Genes and Genomes (KEGG) analysis of DEGs and DAMs at 6 W showed the 10 significantly enriched pathways, including 2 up‐regulated pathways, glucosinolate biosynthesis and 2‐0xocarboxylic acid metabolism, and 8 down‐regulated pathways, Ubiquinone and other terpenoid‐quinone biosynthesis, Cutin, suberine and wax biosynthesis, Fatty acid biosynthesis, Flavonoid biosynthesis, Phenylpropanoid biosynthesis, Tyrosine metabolism, Pyruvate metabolism and Cysteine and methionine metabolism (Figure S4e,f and Figure 6a). According to KEGG database (https://www.genome.jp), the ubiquinone and other terpenoid‐quinone biosynthesis pathway (ko00130) is involved in tocopherol biosynthesis. Tyrosine is one of the precursor for VE biosynthesis, while methionine metabolism serves S‐Adenosyl‐L‐methionine (SAM) as the methyl donor for α‐T biosynthesis. Moreover, tyrosine and methionine are also precursors for the biosynthesis of glucosinolate (Haughn *et al*., 1991; Kroymann *et al*., 2001; Roje, 2006) (Figure S5).

Gene set enrichment analysis (GSEA) indicated that in the ubiquinone and other terpenoid‐quinone biosynthesis pathway, most DEGs (14/15) were down‐regulated in L6WS (Figure 6b). In contrast, in the glucosinolate biosynthesis pathway, 25 out of 28 DEGs up‐regulated (Figure 6c). Heatmap showed changes in gene expression in the biosynthesis pathways of VE and glucosinolate (Figure S4g,h). qRT‐PCR analyses further confirmed the RNA‐seq data. For example, *BnaC06.VTE3* and *BnaC09.VTE5* were down‐regulated in seeds at 6 W (Figure 6d). The expression of several glucosinolate biosynthesis regulating genes, such as *BnaA04.CYP83A1*, *BnaC04.CYP83A1*, *BnaC04.MAM1*, *BnaA05.leuC*, *BnaC04.leuC*, *BnaC08.P45083B1*, *BnaA06.CYP79F1* and *BnaC07.UGT74B1* were integrally up‐regulated (Figure 6d). Interestingly, both DEGs and DAMs significantly enriched in the glucosinolate biosynthesis were up‐regulated in group‐L (Figure 6e and Figure S4h), indicating the reverse regulation mechanism of VE and glucosinolate biosynthesis in rapeseed. In addition, detection of glucosinolate indicated that the total amount of glucosinolate and its components in group‐L were significantly higher than those of group‐H (Figure 6f and Figure S4i).

The contents of VE and glucosinolate in *B. napus* seeds show a highly significant negative correlation (*r* = −0.22, *P* < 0.001) in the natural population (Figure S6). Meanwhile, we found that the glucosinolate content of *BnaC02.VTE4* ^*HapH*^ genotypes was significantly lower than that of *BnaC02.VTE4* ^*HapL*^ genotypes in the associated population (Figure 6g), which was consistent with the distribution of glucosinolate in global rapeseed germplasm resources (Yang *et al*., 2023) (Figure 6h). Concurrently, compared to the negative controls of 8S088 and 8S100, the total glucosinolate content in seed of *BnaC02.VTE4* ^*HapH*^ complementary lines significantly decreased by 44.32% and 45.70%, respectively (Figure 6i and Figure S7). Our results revealed that *BnaC02.VTE4* ^*HapH*^ positively regulated the biosynthesis of VE and negatively regulated the biosynthesis of glucosinolate.

### Potential breeding utilization of *BnaC02.* *VTE4* ^*HapH*^

**Figure 7:**
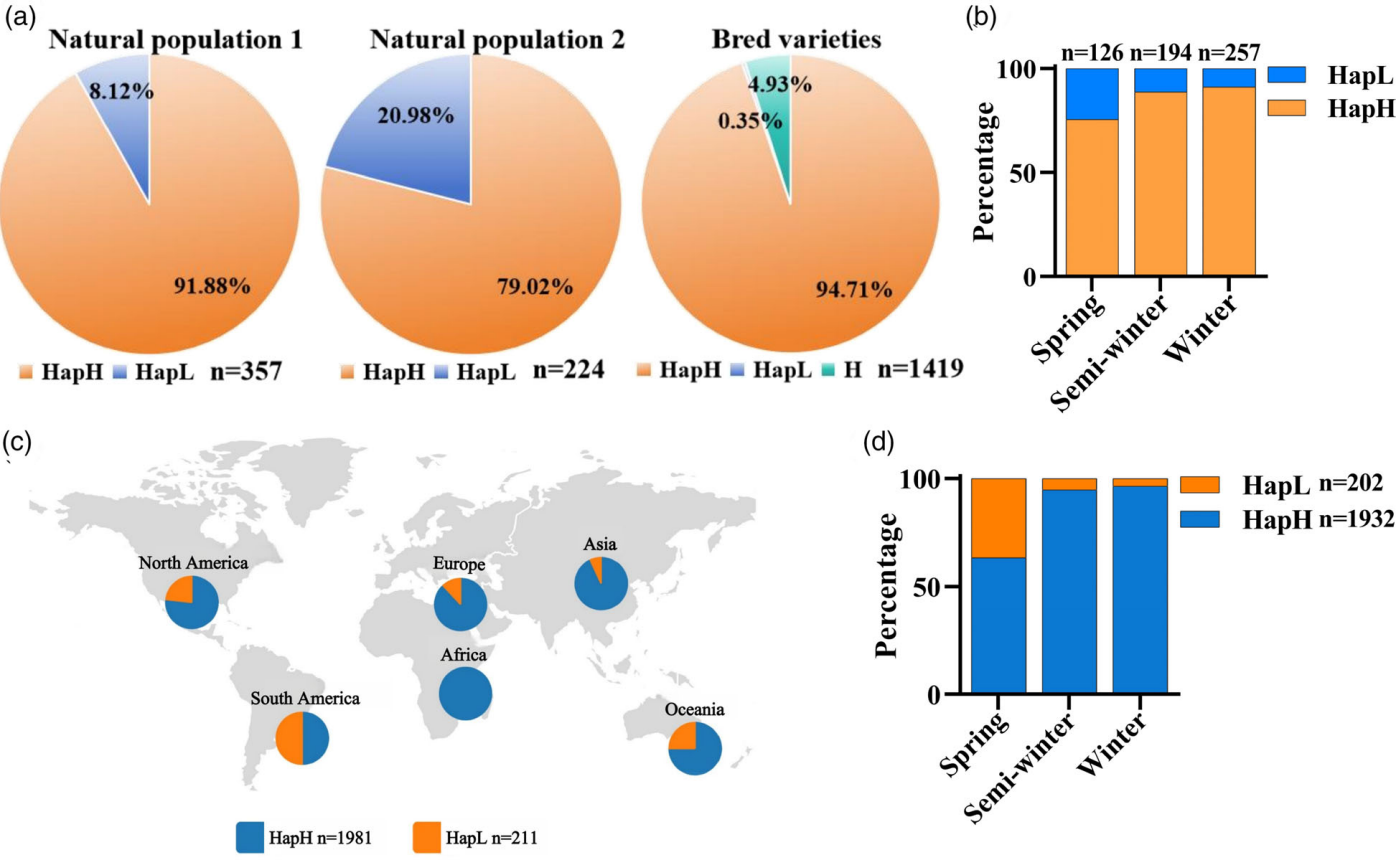
Distribution of *BnaC02.VTE4* ^*HapH*^ and *BnaC02.VTE4* ^*HapL*^ in *B. napus* germplasms and varieties. (a) Distribution of *BnaCO2.VTE4* ^*HapH*^ and *BnaCO2.VTE4* ^*HapL*^ in natural populations and bred varieties. (b) Distribution proportion of *BnaCO2.VTE4* ^*HapH*^ and *BnaCO2.VTE4* ^*HapL*^ alleles in different ecotypes of natural population 1 and 2. (c, d) The analysis of global geographic (c) and ecological distribution (d) of *BnaCO2.VTE4* ^*HapH*^ and *BnaCO2.VTE4* ^*HapL*^ alleles using the germplasms from BnIR. ‘HapH’, homozygote of *BnaCO2.VTE4* ^*HapH*^ genotype; ‘HapL’, homozygote of *BnaCO2.VTE4* ^*HapL*^; ‘H’, represents heterozygote. The pie and bar chart size were proportional to the number of accessions.

Based on the functional variation site of *BnaC02.VTE4*, we developed a KASP (Kompetitive Allele‐Specific PCR) molecular marker C02Vte4 (Table S6). The marker was further used for genetic analysis of three rapeseed populations, including 357 global *B. napus* germplasm resources (Hu *et al*., 2022), 224 *B. napus* germplasms from the association population by Wu *et al*. (2019), and 1419 varieties collected from the winter ecotype regional trials in China. In the association populations, HapH genotype frequency accounted for 91.88% and 79.02%, respectively. However, the proportion of HapH genotype frequency in the bred varieties reached 94.71%, and the proportion of heterozygotes was 4.93%, while that of HapL genotype frequency was only 0.35% (Figure 7a). According to ecotype of *B. napus* in natural populations above, the HapH genotype frequency was present in 75.40% in spring ecotype, increased to 88.66% and 91.05% in semi‐winter and winter ecotype (Figure 7b). Moreover, BnIR showed that the distribution frequency of HapH genotype increased significantly from spring ecotypes to semi‐winter and winter ecotypes in 2192 rapeseed germplasms distributed worldwide (Figure 7c). Generally, HapH genotype has been gradually domesticated in *B. napus* breeding, especially in semi‐winter and winter *B. napus* (Figure 7d).

## Discussion

### The alternative splicing of *BnaC02.VTE4* induced by a point mutation caused the variation of α‐tocopherol content in rapeseed

Vitamin E serves as a source of dietary nutrition (Böhm, 2018). α‐T and γ‐T are the two most prominent VE components in rapeseed, with α‐T being the most active nutrient component of VE (Goffman and Becker, 2002). The conversion from γ‐T to α‐T by the rate‐limiting enzyme γ‐TMT is the final step in the biosynthesis of tocopherols in plants. *Arabidopsis* seeds introduced with the *BnaA.VTE4.a1* gene, which encodes γ‐TMT in *B. napus*, had a 50‐fold increase in α‐T (Endrigkeit *et al*., 2009). *Bna.C02.VTE4* has been re‐mapped in rapeseed through GWAS (Huang *et al*., 2023). Our study identified VE‐associated QTLs through GWAS and excavated the target gene *BnaC02.VTE4* (*BnaC02G197500ZS*) of qVE.C02. A point mutation from A to G occurred at the 3′ splicing site of the second intron of *BnaC02.VTE4*, resulting in an alternative splicing, causing the loss of Ubie_methyltran domain of *VTE4* in rapeseed, finally leading to the differences of VE and α‐T contents in the association population. Consistent with previous studies (Valentin *et al*., 2006), the VE content increased gradually during seed mature in rapeseed, and there were significant differences between the *BnaC02.VTE4* ^*HapH*^ and *BnaC02.VTE4* ^*HapL*^ haplotypes at the 4th week after pollination (Figure 5b). The expression of *BnaC02.VTE4* in the *BnaC02.VTE4* ^*HapH*^ accessions differed significantly from that in the *BnaC02.VTE4* ^*HapL*^ material (Figure 5a). Moreover, there was significant difference in VE content of pod coats between the two genotypes after 5 W (Figure 5b). The regulatory mechanism of VE transport from pod coats to seeds needs further study.

### *BnaC02.VTE4* regulates VE and glucosinolate accumulation by affecting the metabolic direction of tyrosine and methionine

**Figure 8:**
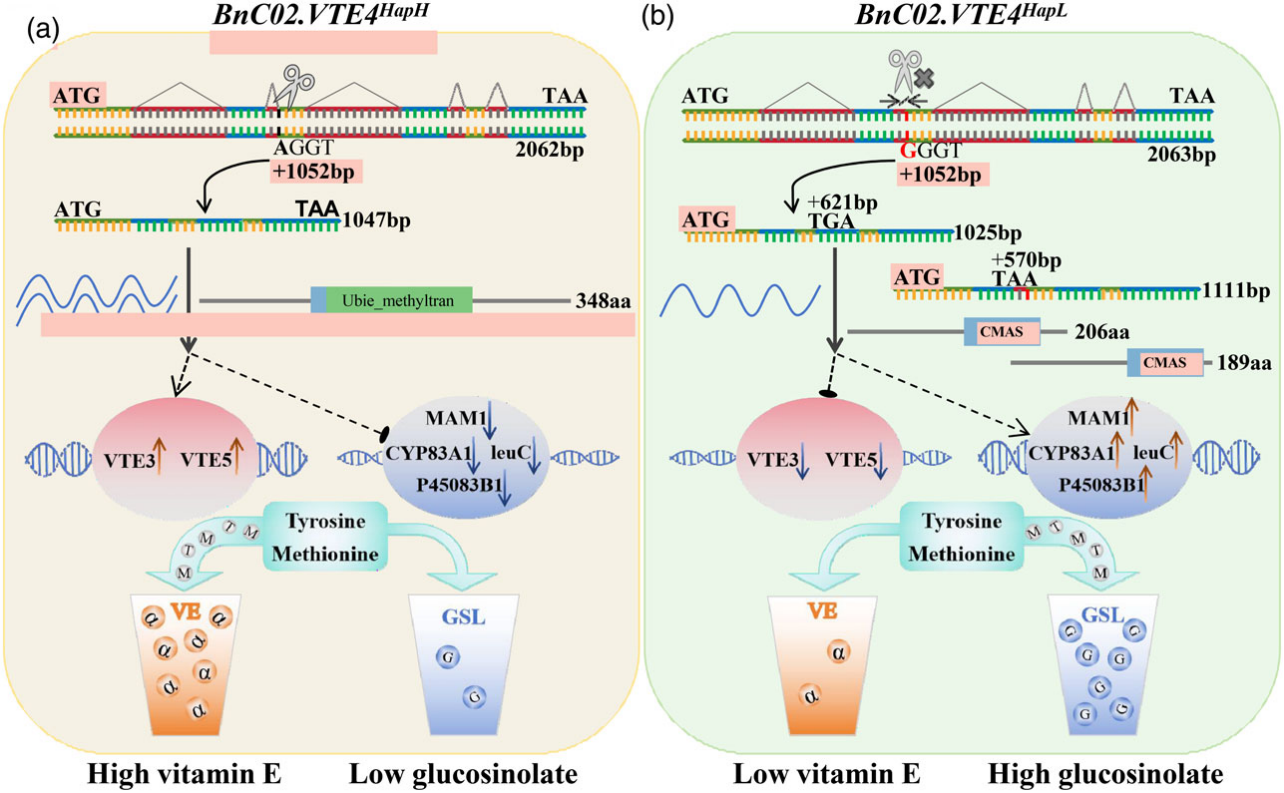
Working mechanism of *BnaC02.VTE4* in the regulation of VE and glucosinolate biosynthesis. (a) The working model of *BnaC02.VTE4* ^*HapH*^, which promoted the conversion of tyrosine and methionine to α‐T. (b) The working model of *BnaC02.VTE4* ^*HapL*^, which led to the redirection of metabolic flux from VE biosynthesis to glucosinolate biosynthesis. GSL, glucosinolate.

The function loss of *BnaC02.VTE4* limits the change of γ‐T to α‐T in the VE biosynthesis pathway and reduces the consumption of tyrosine and methyl donor SAM, resulting in excessive accumulation of methionine. Methionine, as the precursor of SAM biosynthesis, is also the key precursor of glucosinolate biosynthesis (Shin *et al*., 2023; Zhang *et al*., 2024) (Figure S5). Therefore, function loss of *BnaC02.VTE4* leads to the activation of the glucosinolate biosynthesis pathway (Figure 8). It can be concluded that *BnaC02.VTE4* regulates the homeostasis of VE and glucosinolate in rapeseed seeds by affecting the metabolic flow of tyrosine and methionine. *BnaC02.VTE4* ^*HapH*^ positively regulate the biosynthesis of tocopherol and negatively regulate the biosynthesis of glucosinolate. Unlike VE content in rapeseed was mainly controlled by the embryo genotype, the biosynthesis of glucosinolate was mainly regulated by maternal genotype (Haughn *et al*., 1991). The further elucidations of the dynamic balance mechanism of VE and glucosinolate will enrich the understanding of the interaction of maternal and embryo genotypes to regulate *B. napus* seed traits.

### *BnaC02.* *VTE4a* ^*HapH*^ has great potential to improve the seed quality in rapeseed

Increasing the content of tocopherols has always been a key goal of rapeseed quality breeding. Previous studies showed that hybrids in rapeseed have higher α‐T content than their parents (Goffman and Becker, 2001). However, through incomplete diallel hybridization, this study proved that VE and α‐T in rapeseed seeds were mainly controlled by embryo genotype and generally did not possess hybrid vigour. Therefore, to achieve the breeding goal of high VE in rapeseed hybrid, it is necessary to improve the VE traits of both parents.

Glucosinolate is a unique secondary metabolite found in Brassicaceae plants. Some of its degradation products have been proven harmful to human health. Reducing the glucosinolate content in seeds is one of the main objectives in rapeseed breeding (Liu *et al*., 2020; Zhou *et al*., 2023). This study reveals the negative regulatory relationship of VE and glucosinolate through *BnaC02.VTE4*. This will be beneficial to the quality improvement of rapeseed by increasing the VE content and reducing the glucosinolate content simultaneously. Previous study in soybean has shown a positive regulation between tocopherols and fatty acids (Chu *et al*., 2023). Our research also found that fatty acid biosynthesis was down‐regulated in group‐L with *BnaC02.VTE4* ^HapL^ accessions. Researches have provided ample evidence that VE can improve the resistance of crops (Luo *et al*., 2021; Salimath *et al*., 2021; Sundararajan *et al*., 2021). In this study, DEGs and DAMs were significantly co‐enriched in the flavonoid pathway and shown a positive regulation with VE content in rapeseed. Flavonoid biosynthesis pathway is widely reported to be metabolic pathways related to crop stress resistance (Jiang *et al*., 2024; Wang *et al*., 2024; Zhao *et al*., 2024; Zhuang *et al*., 2023). This suggests that VE and flavonoid may have a synergistic effect in regulating stress resistance. We speculated that the use of *BnaC02.VTE4* favourable allele could simultaneously improve multiple seed quality traits, such as increasing VE and FA content, reducing glucosinolate content and had the potential to enhance plant resistance to stress. By development and identification of functional marker of *BnaC02.VTE4* in natural populations and inbred materials, the *BnaC02.VTE4* ^HapH^ was found to be domesticated during breeding. *BnaC02.VTE4* ^HapH^ could help rapeseed to achieve the breeding goal of comprehensive improvement of multiple traits.

## Materials and methods

### Plant materials and growth conditions

The 327 rapeseed germplasm accessions in an association population (Ibrahim *et al*., 2021) were planted at the Yangluo experiment base of the Chinese Academy of Agricultural Sciences (OCRI), Wuhan, Hubei, China during the 2019 and 2020 growing seasons. Each accession was planted in three replicate plots in a randomized block design. Twenty accessions with significant difference in VE content from the association population were planted in Wuchang experimental base of OCRI in 2021, which were used for incomplete diallel hybridization (NCII) and were sampled for RNA‐seq and metabolomics. Transgenic lines were planted in the Hanchuan experimental base of OCRI. Each plot was sown at the end of September and harvested at the following May.

### Measurements of VE profiles and glucosinolate content

Vitamin E traits, including α‐T and γ‐T, were measured on the Waters ultra‐high performance liquid chromatography (UPLC) system, and calculated according to a previously published protocol (Xiao *et al*., 2022). The glucosinolate content was determined using Waters UPLC system according to the method of Chinese agricultural industry standard NY/T 1582–2007 with minor modifications (Xiao *et al*., 2022).

### Phenotypic data statistics and sequence analysis

Excel and SPSS 19.0 were employed for statistical analysis. SnapGene 2.3.2 was used for sequence alignment and ORF prediction. Protein sequence alignment and phylogenetic analysis were performed using Multalin (http://multalin.toulouse.inra.fr/multalin/) and MEGA (Tamura *et al*., 2021). Gene domains were predicted on the Pfam website (http://pfam.xfam.org/) and visualized with TBtools (Chen *et al*., 2020).

### Genome‐wide association analysis and identification of candidate genes

Genome‐wide association study was conducted using the mixed linear model (MLM) with a total of 20 131 SNP markers (deletion rate ≤ 0.2, heterozygosity rate ≤ 0.2, minor allele frequency > 0.05 and SNP marker unique matching) in the Tassel 5.0 software (Bradbury *et al*., 2007). Manhattan and Quantile‐Quantile plot (Q‐Q plot) were obtained using the ‘CMplot’ R package (Yin *et al*., 2021). Haploview software (Barrett *et al*., 2005) was used to conduct LD decay analysis of the ±200 kb of significant SNPs. We merged significantly linked SNPs into a loci and identified the candidate genes underlying the block. Functional annotation of all candidate genes was performed using Darmor‐Bzh (Chalhoub *et al*., 2014), BnIR (https://yanglab.hzau.edu.cn/) and NCBI (https://www.ncbi.nlm.nih.gov/).

### Vector construction and transformation

The full‐length fragments of *BnaC02.VTE4* ^*HapH*^ and *BnaC02.VTE4* ^*HapL*^ (including 2.2 kb upstream, 600 bp downstream of the coding region) were amplified using primer sets CP‐F/CP‐R from 8S069 (HapH) and 8S088 (HapL) genomic DNA, respectively, cut with HindIII/BamHI and cloned into Dsred1300 vector which carrying the visual screening red fluorescence protein (DsRed) (Stuitje *et al*., 2003; Zhang *et al*., 2020a) (Figure S8a). The CDS of three *BnaC02.VTE4* transcripts were amplified from cDNA in seeds of 8S069 (HapH‐T) and 8S088 (HapL‐T1, HapL‐T2), respectively, cut with XbaI/BamHI and then ligated into Dsred1300 with a 35S promoter (Figure S8b). Site‐directed mutagenesis fragments of HapH^1052(A‐G)^ (*BnaC02.VTE4* ^*HapH*^ was mutated from A to G at 1052 bp) and HapL^1052(G‐A)^ (*BnaC02.VTE4* ^*HapL*^ was mutated from G to A at 1052 bp) were cloned into Dsred1300 (Figure S8c). The ligation product were transformed into Agrobacterium tumefaciens GV3101 and then into *Arabidopsis atvte4‐4* using the floral‐dip method (Clough and Bent, 1998; Zhang *et al*., 2006). The recombinant plasmids of *BnaC02.VTE4a* ^*HapH*^, HapH^1052(A‐G)^ and HapL^1052(G‐A)^ were introduced into the *B. napus* 8S088 through Agrobacterium‐mediated transformation. The transgenic plants were verified by the vector autofluorescence, and further confirmed by PCR. All primers used for vector construction and identification are listed in Table S6.

### RNA‐seq and data analysis

Total RNA was extracted from seed samples of group‐H (10 accessions of high VE) and group‐L (10 accessions of low VE) strictly self‐crossed plants at the 4 W and 6 W stages in three replicates. Transcriptome sequencing were performed using DNBSEQ Strand‐Specific by BGI TechSolutions Co., Ltd. (BGI‐Tech, Shenzhen, China). Reads were aligned to the *Brassica napus* ZS11 reference genome (Chen *et al*., 2021; Sun *et al*., 2017). RNA‐seq data analysis was performed using Dr.Tom (https://biosys.bgi.com/) platform, and differentially expressed genes were identified using the criteria DESeq2, |log_2_FC| >1 and *Q* value < 0.05.

### Metabolomics profiling and data analysis

Seeds of 10 high VE rapeseed accessions at 4 W (H4WS) and 6 W (H6WS), 10 low VE rapeseed accessions at 4 W (L4WS) and 6 W (L6WS) were sent to BGI TechSolutions Co., Ltd. (BGI‐Tech, Shenzhen, China) for non‐target metabolomics profiling (*n* = 6). The procedure including metabolite extraction, LC–MS analysis, database searches and bioinformatics analysis. Differentially abundant metabolites were identified using the criteria |FC| > 1.2, *Q* value < 0.05 and VIP > 1. All FC values were calculated as L4WS/H4WS and L6WS/H6WS.

### Relative expression analysis via qRT‐PCR

The leaves, stems, buds, flowers, pods from 1 W to 2 W, seeds and pod coats from 3 W to 7 W of six high VE accessions and six low VE accessions were sampled and immediately frozen in liquid nitrogen. Total RNA were extracted using Fast Pure® Universal Plant Total RNA Isolation Kit (Vazyme, Nanjing, China). HiScript® III 1st Strand cDNA Synthesis Kit (Vazyme) was applied for cDNA synthesis. qRT‐PCR was performed using Taq Pro Universal SYBR qPCR Master Mix (Vazyme) on a Roche LightCycler®96 (Roche, Basel, Switzerland). Rapeseed BnaAct7 was used as the reference gene, and primers for qRT‐PCR were listed in Table S6.

## Conflict of interest

The authors declare that they have no competing interests.

## Author contributions

Xinfa Wang and Hanzhong Wang conceived the project. Xiaoling Dun supervised the project and modified the paper. Furong Wang, Lieqiong Kuang, Zelin Xiao and Ze Tian performed the experiments. Furong Wang analysed the data and wrote the paper. All authors contributed to the article and approved the submitted version.

## Supporting information


**Figure S1** Comparison of full‐length sequences of *BnaC02.VTE4* ^*HapH*^ and *BnaC02.VTE4* ^*HapL*^.
**Figure S2** Cis‐acting regulatory element analysis for *BnaC02.VTE4* within 2200 bp upstream of the start codon.
**Figure S3** Conserved Domain Analysis of γ‐TMT from variety species and different haplotypes of *BnaCO2.VTE4*.
**Figure S4** Basic analysis of transcriptome and metabolome.
**Figure S5** Relationship between VE biosynthesis pathway and glucosinolate biosynthesis pathway.
**Figure S6** Correlation analysis using the average of 2‐year phenotypic data for VE and glucosinolate content in seeds in a natural population of 327 *Brassica napus*.
**Figure S7** Seeds glucosinolate component contents in the complementary transgenic lines of rapeseed.
**Figure S8** Schematic diagram of the vector structure constructed in this study.

**Table S1** The contents of VE and its components in 12 inbred lines and their 13 F1 crosses of rapeseed seeds.

**Table S2** Mid‐parent heterosis analysis of VE‐related traits in rapeseed seeds.

**Table S3** Phenotypic characteristics of VE‐related traits in the *Brassica napus* associated population.

**Table S4** Significant SNP identified by GWAS related to VE, α‐T and γ‐T contents, and α/γ ratio.

**Table S5** Candidate genes surrounding peak SNPs.

**Table S6** The primers used in this study.

## Data Availability

Supplementary data for this article can be found online. RNA‐seq and metabolomics data of 4 W and 6 W developing seeds of group‐H and group‐L have been deposited into the National Genomics Data Center (https://ngdc.cncb.ac.cn/gsa/) database under the GSA Bioproject, accession no. PRJCA032597.
