# Supplementary material for: An alternative splicing caused by a natural variation in BnaC02.VTE4 gene affects vitamin E and glucosinolate content in rapeseed (Brassica napus L.)
Source: Plant Biotechnol J. 2025 Feb 4;23(5):1535–47. doi: 10.1111/pbi.14603 (PMC12018824; doi:10.1111/pbi.14603)
Supplement: Supplementary file 1 — Figure S1 Comparison of full‐length sequences of BnaC02.VTE4 HapH and BnaC02.VTE4 HapL . Figure S2 Cis‐acting regulatory element analysis for BnaC02.VTE4 within 2200 bp upstream of the start codon. Figure S3 Conserved Domain Analysis of γ‐TMT from variety species and different haplotypes of BnaCO2.VTE4. Figure S4 Basic analysis of transcriptome and metabolome. Figure S5 Relationship between VE biosynthesis pathway and glucosinolate biosynthesis pathway. Figure S6 Correlation analysis using the average of 2‐year phenotypic data for VE and glucosinolate content in seeds in a natural population of 327 Brassica napus. Figure S7 Seeds glucosinolate component contents in the complementary transgenic lines of rapeseed. Figure S8 Schematic diagram of the vector structure constructed in this study. [file PBI-23-1535-s003.docx]

(**a**)


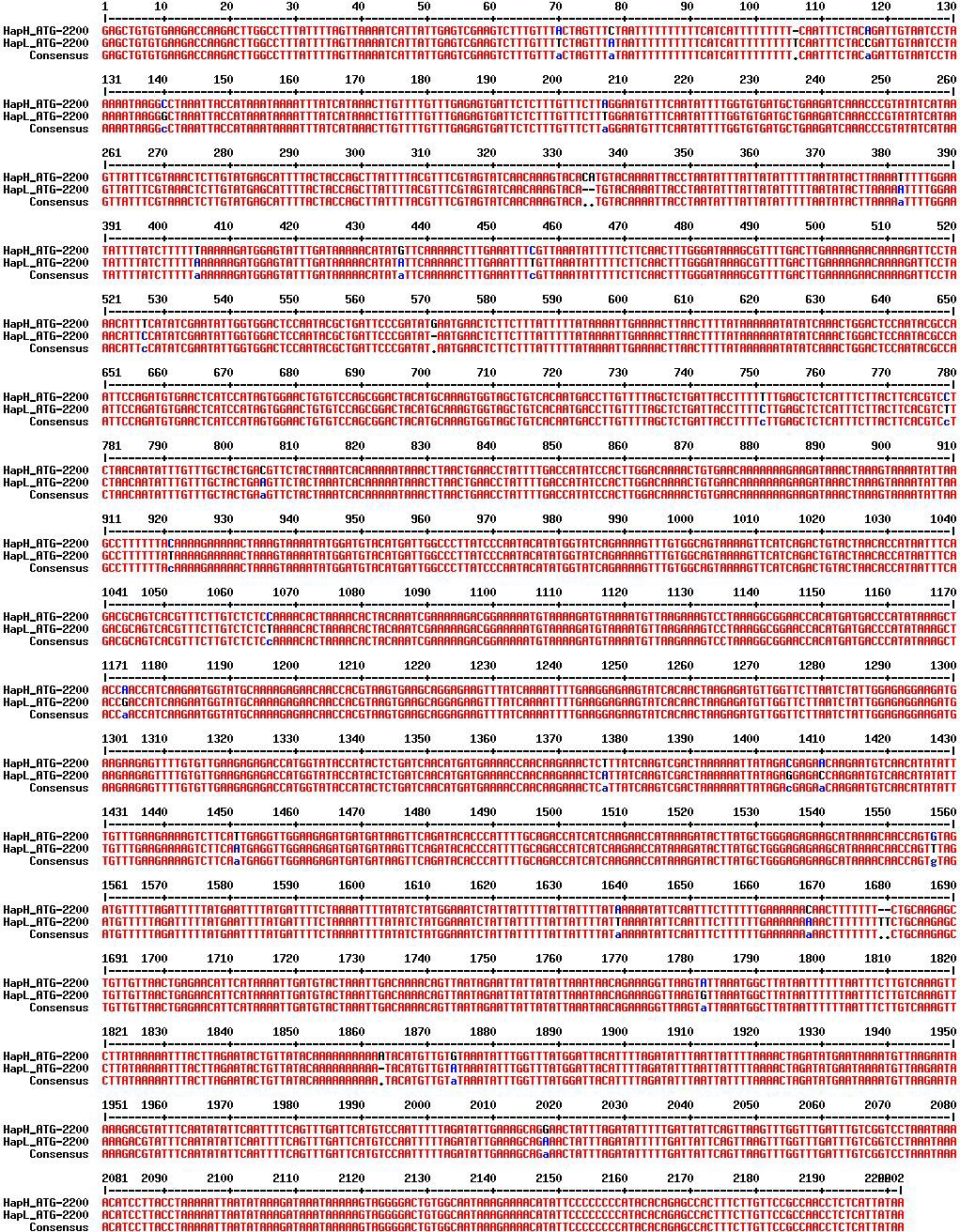


(**b**)


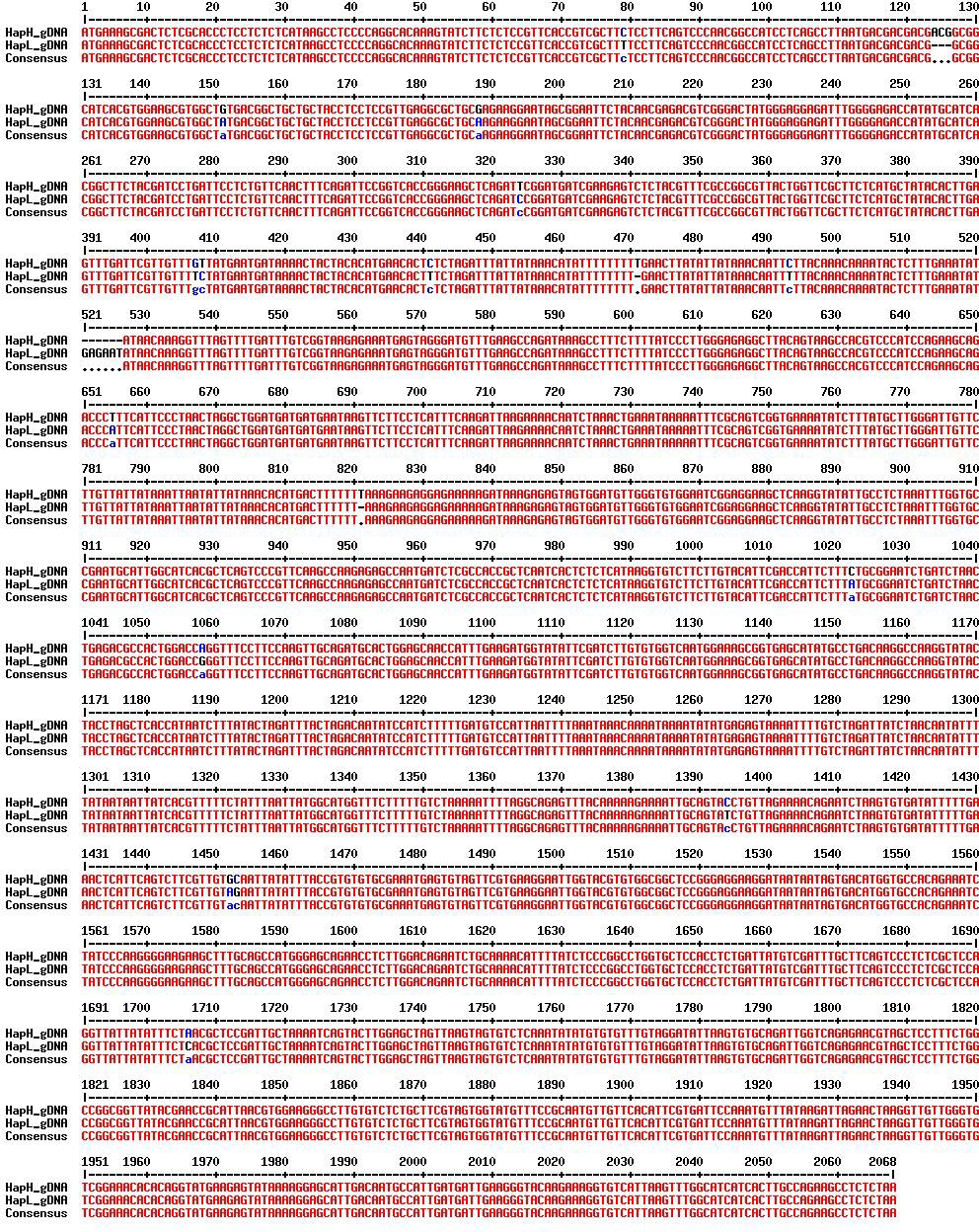


(**c**)


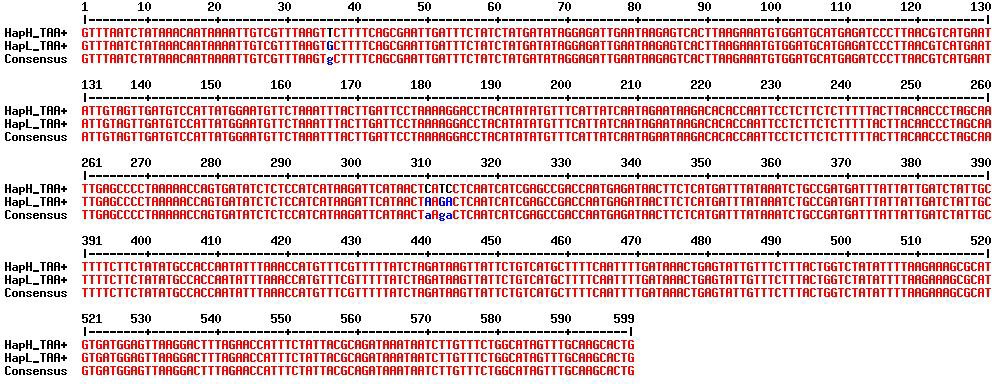


**Fig. S1 Comparison of full-length sequences of *BnaC02.VTE4HapH* and *BnaC02.VTE4HapL*.**

(**a**) 2200bp upstream of the start codon. (**b**) Coding region. (**c**) 599bp downstream of the end codon.


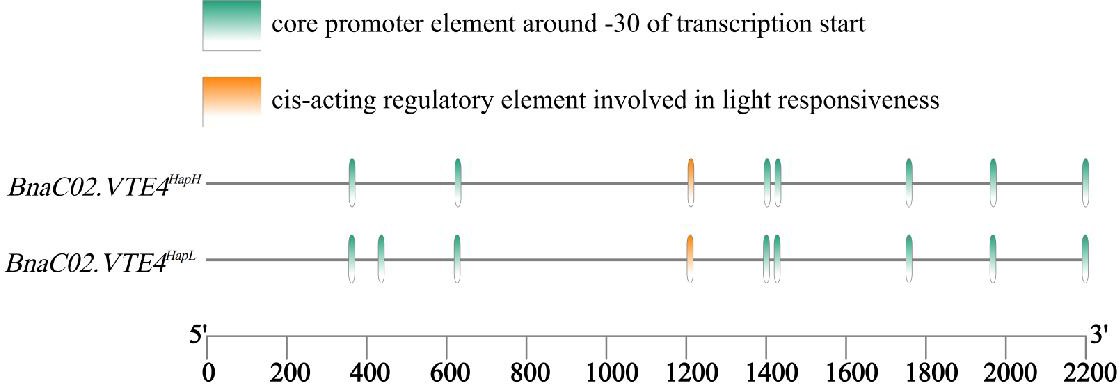


**Fig. S2 Cis-acting regulatory element analysis for *BnaC02.VTE4* within 2200bp upstream of the start codon.**


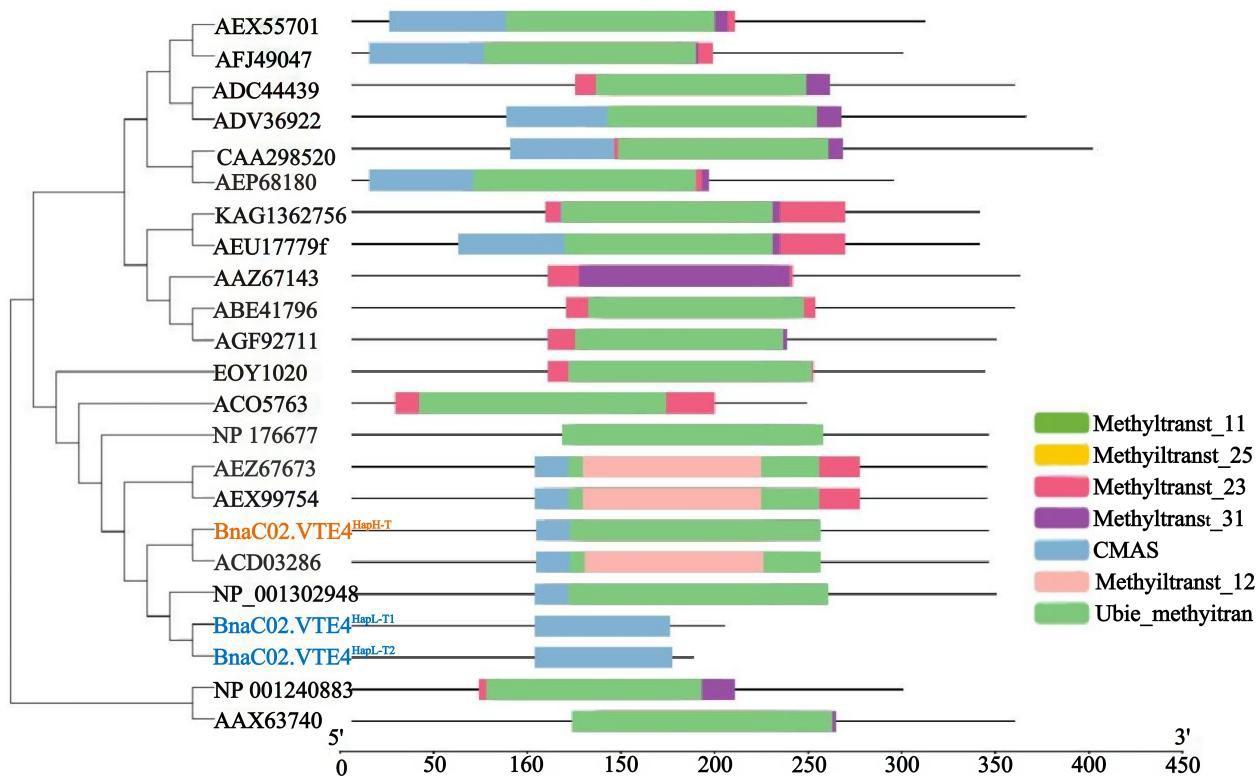


**Fig. S3 Conserved Domain Analysis of γ-TMT from variety species and different haplotypes of *BnaCO2.VTE4*.**


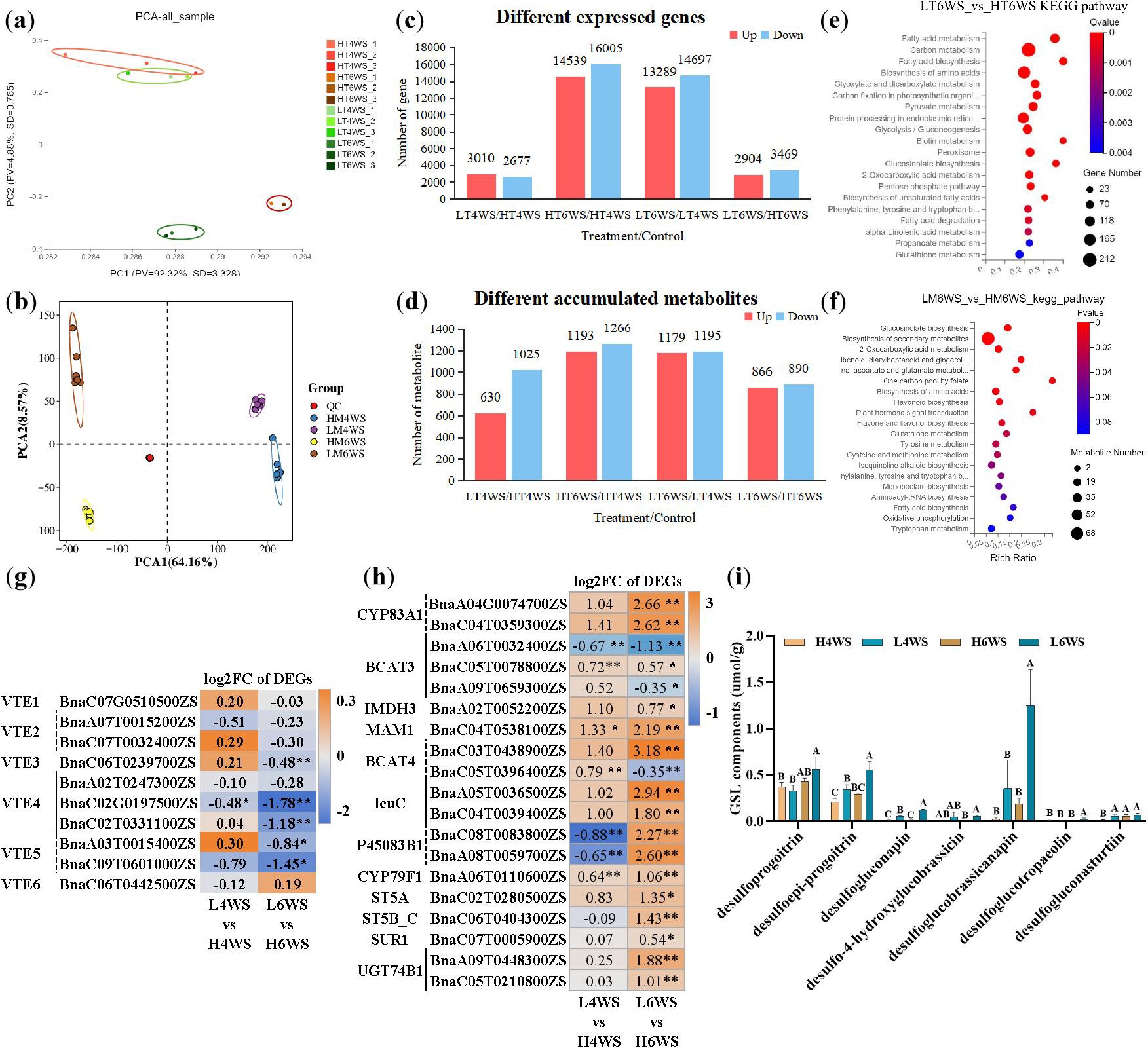


**Fig. S4 Basic analysis of transcriptome and metabolome.** (**a, b**) Principal Component Analysis (PCA) on RNA-seq and metabolism data. (**c, d**) The statistical results of significant DEGs and DAMs. (**e, f**) KEGG pathway enrichment analysis of DEGs and DAMs between group-H and group-L seeds at 6W. (**g, h**) Heatmaps of log2FC (fold changes) of VE and glucosinolate biosynthetic related genes at 4W and 6W seeds of group-H and group-L. (**i**) The various glucosinolate subclass contents in 4W and 6W seeds of group-H and group-L.


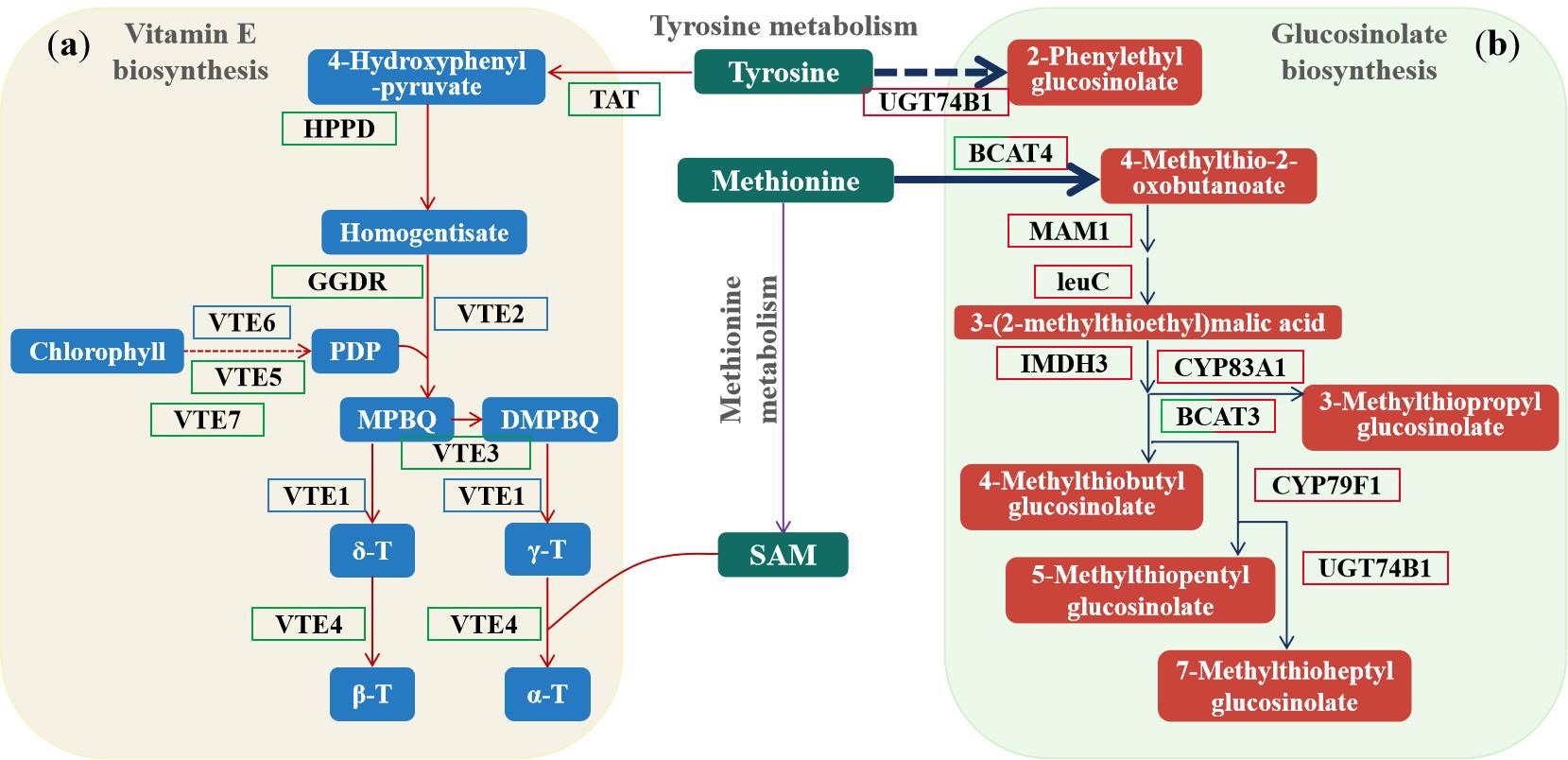


**Fig. S5 Relationship between VE biosynthesis pathway and glucosinolate biosynthesis pathway.** (**a**) VE biosynthesis pathway. (**b**) Glucosinolate biosynthesis pathway.


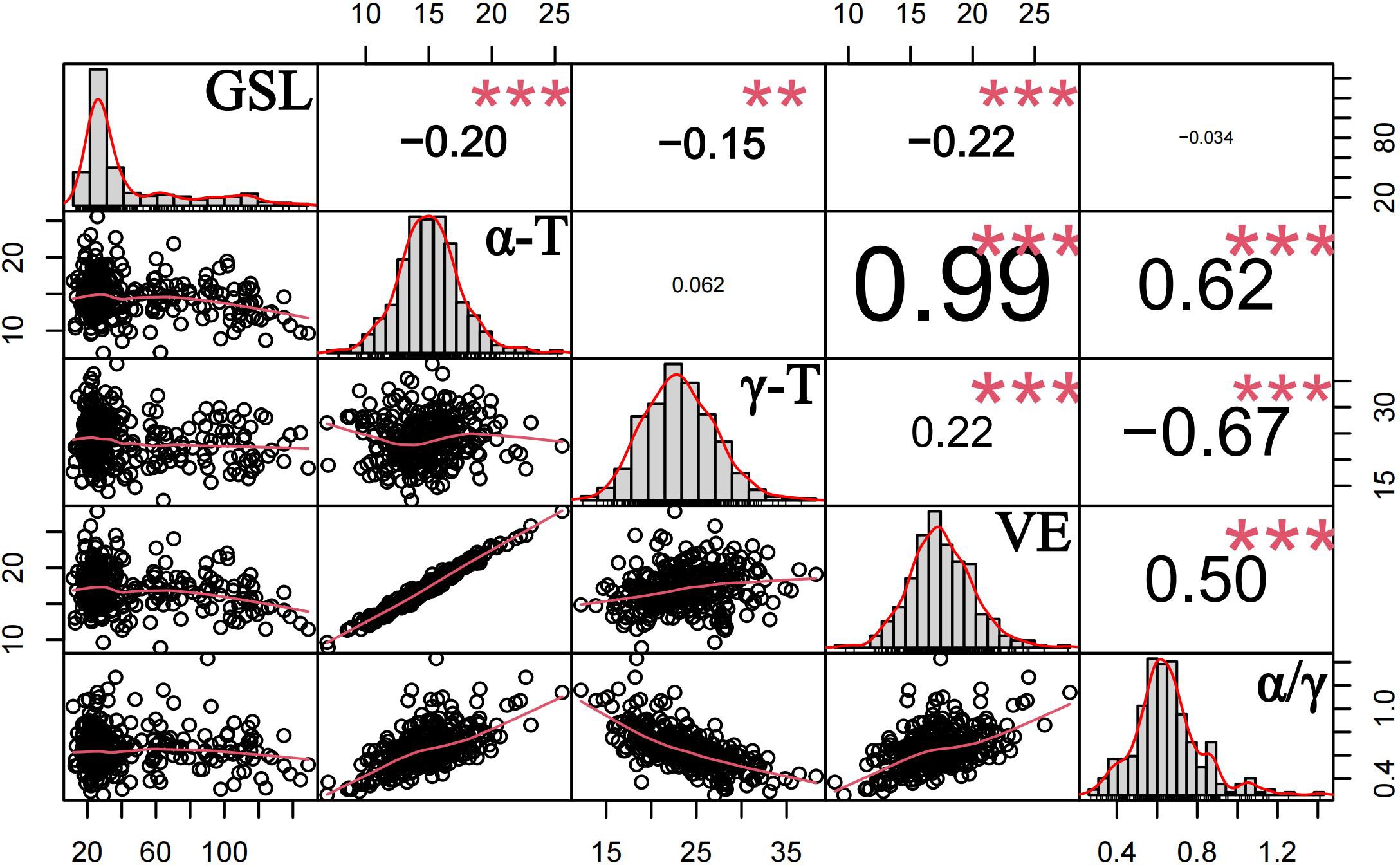


**Fig. S6 Correlation analysis using the average of two-year phenotypic data for VE and glucosinolate content in seeds in a natural population of 327 *B. napus*.**


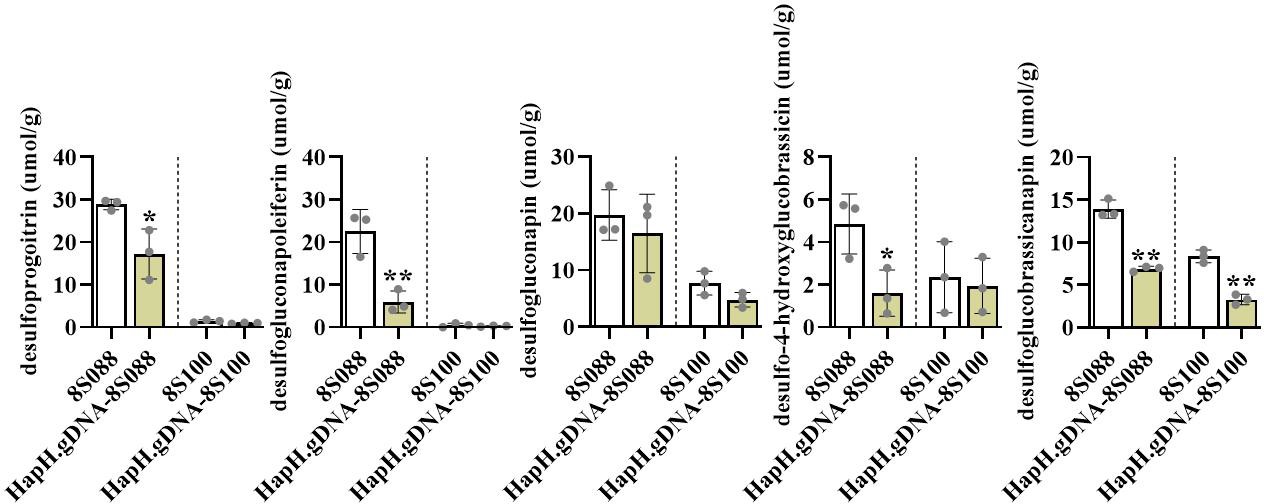


**Fig. S7 Seeds glucosinolate component contents in the complementary transgenic lines of rapeseed.**


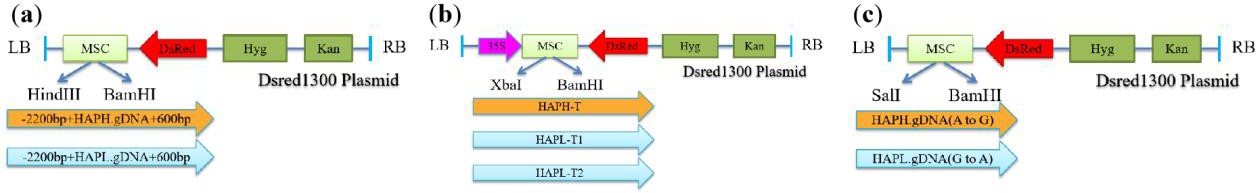


**Fig. S8 Schematic diagram of the vector structure constructed in this study.** (**a**) Full-length complementary vector. (**b**) Overexpression vector. (**c**) Site-directed mutagenesis vector.
