# Supplementary material for: An alternative splicing caused by a natural variation in BnaC02.VTE4 gene affects vitamin E and glucosinolate content in rapeseed (Brassica napus L.)
Source: Plant Biotechnol J. 2025 Feb 4;23(5):1535–47. doi: 10.1111/pbi.14603 (PMC12018824; doi:10.1111/pbi.14603)
Supplement: Supplementary file 2 — Table S1 The contents of VE and its components in 12 inbred lines and their 13 F1 crosses of rapeseed seeds. [file PBI-23-1535-s005.docx]

Table S1 The contents of VE and its components in 12 inbred lines and their 13 F_1_ crosses of rapeseed seeds† Group Accession line VE （mg/100g) α-T （mg/100g)γ-T （mg/100g) α/γ Ratio

| 8S166 | H01 | 28.79±4.01 | 27.33±4.11 | 14.69±0.97 | 1.89±0.41 |
| --- | --- | --- | --- | --- | --- |
| 8S052 | H02 | 24.90±1.15 | 22.33±1.05 | 25.72±1.53 | 0.87±0.04 |
| _H_P 8S180 | H03 | 24.93±0.59 | 23.35±0.72 | 15.73±1.51 | 1.50±0.19 |
| 8S226 | H04 | 20.25±0.27 | 18.67±0.41 | 15.77±1.39 | 1.20±0.14 |
| 8S137 | H05 | 20.52±1.92 | 19.40±2.01 | 11.14±1.02 | 1.76±0.33 |
| 8S069 | H06 | 28.18±0.53 | 25.48±0.54 | 27.04±0.16 | 0.94±0.03 |
| 8S090 | L01 | 9.71±1.12 | 7.85±1.09 | 18.56±2.40 | 0.43±0.08 |
| 8S154 | L02 | 10.41±1.23 | 7.10±0.98 | 33.10±2.71 | 0.21±0.02 |
| _L_P 8S088 | L03 | 13.03±0.78 | 10.43±1.03 | 26.02±3.00 | 0.41±0.09 |
| 8S268 | L04 | 13.64±1.61 | 12.18±1.46 | 14.60±1.52 | 0.84±0.01 |
| 8S313 | L05 | 13.18±0.42 | 11.28±0.52 | 18.99±1.42 | 0.60±0.07 |
| 8S040 | L06 | 13.33±0.44 | 10.59±0.53 | 27.38±0.91 | 0.39±0.03 |
| F ^+^ (^H^P×^L^P) 8S166×8S154 | H01×L02 | 16.73±0.44 | 15.03±0.40 | 17.06±1.04 | 0.88±0.04 |
| - L H  F_1_ ( P× P) 8S154×8S166 | L02×H01 | 17.64±1.54 | 15.34±0.93 | 22.98±1.22 | 0.67±0.03 |
| + H L  F_1_ ( P× P) 8S166×8S313 | H01×L05 | 21.65±0.94 | 19.81±1.43 | 18.43±0.80 | 1.08±0.03 |
| - L H  F_1_ ( P× P) 8S313×8S166 | L05×H01 | 13.30±0.74 | 11.82±0.54 | 14.83±1.04 | 0.80±0.03 |
| + H L  F_1_ ( P× P) 8S052×8S154 | H02×L02 | 15.50±0.66 | 13.51±0.90 | 19.92±2.40 | 0.69±0.13 |
| - L H  F_1_ ( P× P) 8S154×8S052 | L02×H02 | 16.15±0.47 | 13.87±0.46 | 22.76±0.02 | 0.61±0.02 |
| + H L  F_1_ ( P× P) 8S052×8S088 | H02×L03 | 17.82±0.99 | 15.59±0.71 | 22.29±1.56 | 0.70±0.03 |
| - L H  F_1_ ( P× P) 8S088×8S052 | L03×H02 | 15.12±0.40 | 13.42±0.36 | 17.01±1.03 | 0.79±0.04 |
| + H L  F_1_ ( P× P) 8S052×8S268 | H02×L04 | 19.56±1.09 | 17.75±0.81 | 18.11±1.27 | 0.98±0.03 |
| - L H  F_1_ ( P× P) 8S268×8S052 | L04×H02 | 17.26±1.50 | 16.02±0.97 | 12.41±0.66 | 1.29±0.06 |
| + H L  F_1_ ( P× P) 8S180×8S090 | H03×L01 | 12.78±0.56 | 11.65±0.84 | 11.32±0.49 | 1.03±0.03 |
| - L H  F_1_ ( P× P) 8S090×8S180 | L01×H03 | 12.06±0.80 | 10.86±0.65 | 12.02±2.15 | 0.93±0.13 |
| + H L  F_1_ ( P× P) 8S180×8S154 | H03×L02 | 17.19±0.75 | 15.41±1.11 | 17.73±0.77 | 0.87±0.03 |
| - L H  F_1_ ( P× P) 8S154×8S180 | L02×H03 | 20.71±1.81 | 18.21±1.11 | 24.94±1.32 | 0.73±0.04 |
| + H L  F_1_ ( P× P) 8S180×8S088 | H03×L03 | 19.83±1.05 | 17.92±0.80 | 19.02±2.59 | 0.95±0.08 |
| - L H  F_1_ ( P× P) 8S088×8S180 | L03×H03 | 22.65±0.77 | 20.34±0.69 | 23.11±0.79 | 0.88±0.00 |
| + H L  F_1_ ( P× P) 8S226×8S154 | H04×L02 | 14.81±1.29 | 12.81±0.78 | 20.02±1.06 | 0.64±0.03 |
| - L H  F_1_ ( P× P) 8S154×8S226 | L02×H04 | 16.80±0.44 | 14.07±0.37 | 27.37±1.66 | 0.51±0.02 |
| + H L  F_1_ ( P× P) 8S226×8S088 | H04×L03 | 17.87±0.47 | 15.39±0.41 | 24.76±1.51 | 0.62±0.03 |
| - L H  F_1_ ( P× P) 8S088×8S226 | L03×H04 | 17.07±0.74 | 14.93±1.08 | 21.45±0.93 | 0.70±0.03 |
| + H L  F_1_ ( P× P) 8S137×8S268 | H05×L04 | 18.18±1.01 | 17.03±0.78 | 11.50±0.81 | 1.48±0.05 |
| - L H  F_1_ ( P× P) 8S268×8S137 | L04×H05 | 14.90±2.66 | 13.63±2.22 | 12.65±4.34 | 1.15±0.22 |
| + H L  F_1_ ( P× P) 8S137×8S313 | H05×L05 | 14.32±1.25 | 13.46±0.82 | 8.61±0.46 | 1.56±0.08 |
| - L H  F_1_ ( P× P) 8S313×8S137 | L05×H05 | 15.19±0.73 | 13.66±0.58 | 15.31±1.65 | 0.90±0.07 |
| + H L  F_1_ ( P× P) 8S069×8S090 | H06×L01 | 18.55±0.81 | 16.08±1.16 | 24.71±1.08 | 0.65±0.03 |
| - L H  F_1_ ( P× P) 8S090×8S069 | L01×H06 | 18.43±0.49 | 16.05±0.42 | 23.84±1.45 | 0.67±0.03 |

1

†Data are presented as mean±SD, n = 3 biological replicates.
