# Supplementary material for: An alternative splicing caused by a natural variation in BnaC02.VTE4 gene affects vitamin E and glucosinolate content in rapeseed (Brassica napus L.)
Source: Plant Biotechnol J. 2025 Feb 4;23(5):1535–47. doi: 10.1111/pbi.14603 (PMC12018824; doi:10.1111/pbi.14603)
Supplement: Supplementary file 3 — Table S2 Mid‐parent heterosis analysis of VE‐related traits in rapeseed seeds. [file PBI-23-1535-s004.docx]

Table S2 Mid-parent heterosis analysis of VE-related traits in rapeseed seeds.

Crosses

H01-L02 H01-L05 H02-L02 H02-L03 H02-L04 H03-L01 H03-L02 H03-L03 H04-L02 H04-L03 H05-L04 H05-L05 H06-L01

Mean

F +

1

F -

1

VE

-14.64

3.19

-12.21

-6.07

1.13

-26.18

-2.72

4.47

-3.41

7.36

6.04

-14.99

-2.10

-4.62

α-T

-12.70

2.64

-8.20

-4.84

2.31

-25.31

1.24

6.12

-0.63

5.76

7.24

-12.25

-3.53

-3.24

γ-T

-28.61

9.43

-32.27

-13.84

-9.12

-34.01

-27.38

-8.89

-18.06

18.46

-9.02

-42.86

8.37

-14.45

α/γ

-16.13

-13.45

28.11

9.54

12.08

6.96

1.56

0.05

-9.24

-22.44

12.20

32.61

-4.99

2.84

VE

-10.01

-36.62

-8.54

-20.29

-10.75

-30.36

17.22

19.34

9.58

2.57

-13.09

-9.82

-2.72

-7.19

α-T

-10.88

-38.76

-5.75

-18.08

-7.64

-30.40

19.60

20.42

9.17

2.59

-14.17

-10.94

-3.70

-6.81

γ-T

-3.82

-11.92

-22.61

-34.26

-37.73

-29.90

2.15

10.68

12.02

2.64

0.10

1.63

4.57

-8.19

α/γ

-36.22

-35.60

12.63

23.76

47.56

-3.41

-14.73

-7.64

-27.64

-12.67

-12.89

-23.68

-2.17

-7.13
