# Supplementary material for: An alternative splicing caused by a natural variation in BnaC02.VTE4 gene affects vitamin E and glucosinolate content in rapeseed (Brassica napus L.)
Source: Plant Biotechnol J. 2025 Feb 4;23(5):1535–47. doi: 10.1111/pbi.14603 (PMC12018824; doi:10.1111/pbi.14603)
Supplement: Supplementary file 4 — Table S3 Phenotypic characteristics of VE‐related traits in the Brassica napus associated population. [file PBI-23-1535-s006.docx]

Table S3 Phenotypic characteristics of VE-related traits in the B. napus associated population

| **Name** | **Trait** | **Year** | **n** | **Min.** | **Max.** | **Mean** | **SD** | **CV** | **Skewness** | **Kurtosis** |
| --- | --- | --- | --- | --- | --- | --- | --- | --- | --- | --- |
| 19VE | VE | 2019 | 312 | 9.00 | 30.00 | 18.01 | 3.07 | 17.05% | 0.44 | 1.02 |
| 20VE | VE | 2020 | 316 | 8.51 | 25.69 | 16.14 | 2.75 | 17.04% | 0.25 | 0.21 |
| 19α-T | α-T | 2019 | 312 | 7.30 | 27.40 | 15.46 | 2.97 | 19.21% | 0.45 | 1.32 |
| 20α-T | α-T | 2020 | 316 | 6.51 | 23.70 | 13.94 | 2.77 | 19.87% | 0.24 | 0.43 |
| 19γ-T | γ-T | 2019 | 312 | 11.40 | 43.30 | 25.63 | 5.14 | 20.05% | 0.16 | 0.43 |
| 20γ-T | γ-T | 2020 | 316 | 9.51 | 36.56 | 21.97 | 4.51 | 20.53% | 0.28 | 0.24 |
| 19α/γ | α/γ Ratio | 2019 | 312 | 0.25 | 1.34 | 0.63 | 0.03 | 4.76% | 0.90 | 1.72 |
| 20α/γ | α/γ Ratio | 2020 | 316 | 0.23 | 2.30 | 0.67 | 0.05 | 7.46% | 2.09 | 11.02 |

**.**

**h2**

0.81

0.83

0.69

-
