# Supplementary material for: An alternative splicing caused by a natural variation in BnaC02.VTE4 gene affects vitamin E and glucosinolate content in rapeseed (Brassica napus L.)
Source: Plant Biotechnol J. 2025 Feb 4;23(5):1535–47. doi: 10.1111/pbi.14603 (PMC12018824; doi:10.1111/pbi.14603)
Supplement: Supplementary file 5 — Table S4 Significant SNP identified by GWAS related to VE, α‐T and γ‐T contents, and α/γ ratio. [file PBI-23-1535-s002.docx]

Table S4 Significant SNP identified by GWAS related to VE, α-T and γ-T contents, and α/γ

| **Cluster Trait** | **Marker** | **Chr** | **Pos** | **P** | **-LOG10(Pvalue)** | **PVE (%)** |
| --- | --- | --- | --- | --- | --- | --- |
| qVE.A01 19α/γ | Bn-A01-p20890858 | A01 | 17693741 | 2.81E-06 | 5.55 | 8.83% |
| 19α/γ | Bn-A01-p20933189 | A01 | 17737678 | 1.43E-06 | 5.84 | 10.41% |
| qVE.A02 20α/γ | Bn-A02-p6391751 | A02 | 3579819 | 4.33E-05 | 4.36 | 6.67% |
| qVE.A07 20VE | Bn-A07-p1210412 | A07 | 874226 | 3.37E-05 | 4.47 | 7.58% |
| 20VE | seq-new-rs38730 | C02 | 11638002 | 2.01E-07 | 6.70 | 11.76% |
| 20α-T | seq-new-rs38730 | C02 | 11638002 | 1.98E-08 | 7.70 | 13.61% |
| 19VE | seq-new-rs38730 | C02 | 11638002 | 9.23E-10 | 9.03 | 15.59% |
| 19α-T | seq-new-rs38730 | C02 | 11638002 | 4.03E-11 | 10.39 | 18.17% |
| 20VE | seq-new-rs25934 | C02 | 11657000 | 1.20E-07 | 6.92 | 11.05% |
| 19VE | seq-new-rs25934 | C02 | 11657000 | 4.47E-08 | 7.35 | 11.45% |
| 20α-T | seq-new-rs25934 | C02 | 11657000 | 2.17E-08 | 7.66 | 12.40% |
| 19α-T | seq-new-rs25934 | C02 | 11657000 | 3.94E-09 | 8.40 | 13.42% |
| 20VE | seq-new-rs25964 | C02 | 11658359 | 1.04E-06 | 5.98 | 10.99% |
| qVE.C02 20α-T | seq-new-rs25964 | C02 | 11658359 | 1.00E-07 | 7.00 | 12.91% |
| 19VE | seq-new-rs25964 | C02 | 11658359 | 4.52E-08 | 7.34 | 12.99% |
| 19α-T | seq-new-rs25964 | C02 | 11658359 | 2.62E-09 | 8.58 | 15.36% |
| 20VE | seq-new-rs29140 | C02 | 11738438 | 1.06E-05 | 4.98 | 8.74% |
| 20α-T | seq-new-rs29140 | C02 | 11738438 | 1.80E-06 | 5.75 | 10.14% |
| 19VE | seq-new-rs29140 | C02 | 11738438 | 1.93E-07 | 6.71 | 11.75% |
| 19α-T | seq-new-rs29140 | C02 | 11738438 | 1.38E-08 | 7.86 | 13.91% |
| 20VE | seq-new-rs31838 | C02 | 11897577 | 2.90E-09 | 8.54 | 14.21% |
| 20α-T | seq-new-rs31838 | C02 | 11897577 | 2.54E-10 | 9.60 | 16.24% |
| 19VE | seq-new-rs31838 | C02 | 11897577 | 2.46E-10 | 9.61 | 16.10% |
| 19α-T | seq-new-rs31838 | C02 | 11897577 | 1.14E-11 | 10.94 | 18.71% |
