# Supplementary material for: An alternative splicing caused by a natural variation in BnaC02.VTE4 gene affects vitamin E and glucosinolate content in rapeseed (Brassica napus L.)
Source: Plant Biotechnol J. 2025 Feb 4;23(5):1535–47. doi: 10.1111/pbi.14603 (PMC12018824; doi:10.1111/pbi.14603)
Supplement: Supplementary file 6 — Table S5 Candidate genes surrounding peak SNPs. [file PBI-23-1535-s007.docx]

Table S5 Candidate genes surrounding peak SNPs.

| **Gene alias** | **Locus** | **A.thaliana homologue** | **Functional description** |
| --- | --- | --- | --- |
| *BnaC02g16010D* | chrC02:11623538-11632987 | *AT1G14800.1* | Nucleic acid-binding, OB-fold-like protein |
| *BnaC02g16020D* | chrC02:11641672-11643299 | *AT5G50830.1* | unknown protein |
| *BnaC02g16030D* | chrC02:11650211-11652702 | *AT5G50770.1* | hydroxysteroid dehydrogenase 6 (HSD6) |
| *BnaC02g16040D* | chrC02:11657343-11658730 | *AT5G50710.1* | unknown protein |
| *BnaC02g16050D* | chrC02:11730646-11732683 | *AT5G50520.1* | Major facilitator superfamily protein |
| *BnaC02g16060D* | chrC02:11736741-11737350 | *AT5G50115.1* | CONTAINS InterPro DOMAIN/s |
| *BnaC02g16070D* | chrC02:11745682-11746793 | *AT5G50460.1* | secE/sec61-gamma protein transport protein |
| *BnaC02g16080D BnaC02g16090D* | chrC02:11751376-11752553 chrC02:11757360-11757693 | *AT5G50450.1* | HCP-like superfamily protein with MYND-type zinc finger |
| *BnaC02g16100D* | chrC02:11759662-11761963 | *AT5G50420.1* | O-fucosyltransferase family protein |
| *BnaC02g16110D* | chrC02:11770633-11771886 | *AT5G50410.1* | unknown protein |
| *BnaC02g16120D* | chrC02:11777282-11779558 | *AT4G28090.1* | SKU5 similar 10 |
| *BnaC02g16130D BnaC02g16140D* | chrC02:11783760-11787268 chrC02:11787369-11787631 | *AT5G50360.1* | unknown protein |
| *BnaC02g16150D* | chrC02:11811261-11812589 | *AT5G50200.1* | WOUND-RESPONSIVE 3 (WR3) |
| *BnaC02g16160D* | chrC02:11821880-11826426 | *AT5G49960.1* | unknown protein |
| *BnaC02g16170D* | chrC02:11826918-11827950 | *AT4G39550.1* | Galactose oxidase/kelch repeat superfamily protein |
| *BnaC02g16180D* | chrC02:11828215-11830925 | *AT2G05260.1* | alpha/beta-Hydrolases superfamily protein |
| *BnaC02g16190D* | chrC02:11831221-11832454 | *AT5G61020.1* | evolutionarily conserved C-terminal region 3 (ECT3) |
| *BnaC02g16200D* | chrC02:11835534-11836616 | *AT5G49920.1* | Octicosapeptide/Phox/Bem1p family protein |
| BnaC02g16210D | chrC02:11850257-11853792 | AT5G49890.1 | chloride channel C (CLC-C) |
| BnaC02g16220D | chrC02:11859325-11859996 | AT4G18110.1 | RING/U-box superfamily protein |
| BnaC02g16230D | chrC02:11860891-11861329 | AT2G35280.1 | F-box family protein |
| BnaC02g16240D | chrC02:11864919-11867943 | AT5G49820.1 | CONTAINS InterPro DOMAIN/s |
| BnaC02g16250D BnaC02g16260D | chrC02:11879128-11880981 chrC02:11883492-11891907 | AT5G49810.1 | methionine S-methyltransferase (MMT) |
| BnaC02g16270D | chrC02:11894032-11896047 | AT1G64970.1 | gamma-tocopherol methyltransferase (γ-TMT) |
| BnaC02g16280D | chrC02:11896145-11896356 |  |  |
| *BnaC02g16290D* | chrC02:11899221-11900427 | *AT1G64980.1* | Nucleotide-diphospho-sugar transferases superfamily protein |
