# Supplementary material for: An alternative splicing caused by a natural variation in BnaC02.VTE4 gene affects vitamin E and glucosinolate content in rapeseed (Brassica napus L.)
Source: Plant Biotechnol J. 2025 Feb 4;23(5):1535–47. doi: 10.1111/pbi.14603 (PMC12018824; doi:10.1111/pbi.14603)
Supplement: Supplementary file 7 — Table S6 The primers used in this study. [file PBI-23-1535-s001.docx]

**Table S6 The primers used in this study.**

**Primer name Sequence (5’ to 3’)**

**For PCR Analysis**

pEX14-F TGCTGCTACCTCCTCCGTTG pEX14-R CAAAGCTTCTTCCCCTTGGG

# For qRT-PCR Analysis

BnaAct7 F CTGGAATTGCTGACCGTATGAG BnaAct7 R ATCTGTTGGAAAGTGCTGAGGG BnaCO2.VTE4 F CGCTCCAGGATATTAAGTGT BnaCO2.VTE4 R GCAAGTGATGATGCCAAACT C6.VTE3 F GGGATGCTCTGTCACCGG C6.VTE3 R AAACCAGGCAGCCGCTAG C9.VTE5 F TCATCGGCTCGTCGAGTCG C9.VTE5 R AGACAAGCGCGTAGGCTC A4.CYP83A1 F CACGTGTGGCCACCTTCA A4.CYP83A1 R GCTTGCCTGCACACAACC C4.CYP83A1 F GTGTCCCGCGACGAGAAA C4.CYP83A1 R AGACGCATTCCAGGGCAC C4.MAM1 F TCGCCAAAACCGTGGGAA C4.MAM1 R GACTCCCAAGCCGCCTTT A5.leuC F GACTCCCACACAACCCGG A5.leuC R GGGAGCGTGTTCACGTGA C4.leuC F ACTTTGGTTGCGGGTCGT C4.leuC R GAGCGGAAACACCTCCCC C8.P45083B1 F GTGCCCCGCTATGCATCT C8.P45083B1 R TCCCTGTCGGTAGGCTCC A6.CYP79F1 F ACTTCGCCGGAACACACG A6.CYP79F1 R ATTTGGCCGGTCTGCGAA A9.UGT74B1 F TCTCCGACGGTCACGACT A9.UGT74B1 R AAGGTTTCGGAGCCGTGG C5.UGT74B1 F GCTGAGCAAGTCGGTGGT C5.UGT74B1 R CCTTTGCCACCTCAGCGA

# For gene cloning

PgDNA F GAGCTGTGTGAAGACCAAGACT PgDNA R CAGTGCTTGCAAACTATGCC PcDNA F ATGAAAGCGACTCTCGCACC PcDNA R TTAGAGAGGCTTCTGGCAAG

# For complementary vector construction

CP F acgacggccagtgccaagcttGAGCTGTGTGAAGACCAAGACTTG CP R gaacgatcgggaattggatccCAGTGCTTGCAAACTATGCCAG **For overexpression vector construction**

OE F agcagatctatcgattctagaATGAAAGCGACTCTCGCACC

OE R gaacgatcgggaattggatccTTAGAGAGGCTTCTGGCAAGTGA

# For genetic transformation positive identification

Hyb F1 GTTCGACAGCGTCTCCGACCTGAT Hyb R1 TGTAGTGTATTGACCGATTCCTTGC Hyb F2 CAAGACCTGCCTGAAACCGA

Hyb R2 GAGCATATACGCCCGGAGTC

# Molecular marker

C02Vte4 Fa GAAGGTGACCAAGTTCATGCTACTGAGACGCCACTGGACCA C02Vte4 Fg GAAGGTCGGAGTCAACGGATTTGAGACGCCACTGGACCG C02Vte4 R GGACATCAAAAAGATGGATATTGTCTAG
